# Associations of antidiabetic drugs with diabetic retinopathy in people with type 2 diabetes: an umbrella review and meta-analysis

**DOI:** 10.3389/fendo.2023.1303238

**Published:** 2024-01-03

**Authors:** Luyuan Tan, Zhaonan Wang, Kelvin Okoth, Konstantinos A. Toulis, Alastair K. Denniston, Baldev M. Singh, Francesca L. Crowe, Christopher Sainsbury, Jingya Wang, Krishnarajah Nirantharakumar

**Affiliations:** ^1^ Institute of Applied Health Research, University of Birmingham, Birmingham, United Kingdom; ^2^ Academic Unit of Ophthalmology, Institute of Inflammation and Ageing, University of Birmingham, Birmingham, United Kingdom; ^3^ National Institute for Health and Care Research Birmingham Biomedical Research Centre, Birmingham, United Kingdom; ^4^ Wolverhampton Diabetes Centre, New Cross Hospital, The Royal Wolverhampton National Health Service Trust, Wolverhampton, United Kingdom; ^5^ Research Institute in Healthcare Sciences, Faculty of Science & Engineering, University of Wolverhampton, Wolverhampton, United Kingdom

**Keywords:** type 2 diabetes, antidiabetic medications, diabetic retinopathy, meta-review, diabetic complication

## Abstract

**Background:**

Diabetic retinopathy (DR) is the most frequent complication of type 2 diabetes and remains the leading cause of preventable blindness. Current clinical decisions regarding the administration of antidiabetic drugs do not sufficiently incorporate the risk of DR due to the inconclusive evidence from preceding meta-analyses. This umbrella review aimed to systematically evaluate the effects of antidiabetic drugs on DR in people with type 2 diabetes.

**Methods:**

A systematic literature search was undertaken in Medline, Embase, and the Cochrane Library (from inception till 17th May 2022) without language restrictions to identify systematic reviews and meta-analyses of randomized controlled trials or longitudinal studies that examined the association between antidiabetic drugs and DR in people with type 2 diabetes. Two authors independently extracted data and assessed the quality of included studies using the AMSTAR-2 (A MeaSurement Tool to Assess Systematic Reviews) checklist, and evidence assessment was performed using the GRADE (Grading of recommendations, Assessment, Development and Evaluation). Random-effects models were applied to calculate relative risk (RR) or odds ratios (OR) with 95% confidence intervals (CI). This study was registered with PROSPERO (CRD42022332052).

**Results:**

With trial evidence from 11 systematic reviews and meta-analyses, we found that the use of glucagon-like peptide-1 receptor agonists (GLP-1 RA), sodium-glucose cotransporter-2 inhibitors (SGLT-2i), or dipeptidyl peptidase-4 inhibitors (DPP-4i) was not statistically associated with the risk of DR, compared to either placebo (RR: GLP-1 RA, 0.98, 0.89-1.08; SGLT-2i, 1.00, 95% CI 0.79-1.27; DPP-4i, 1.17, 0.99-1.39) or other antidiabetic drugs. Compared to other antidiabetic drugs, meglitinides (0.34, 0.01-8.25), SGLT-2i (0.73, 0.10-5.16), thiazolidinediones (0.92, 0.67-1.26), metformin (1.15, 0.81-1.63), sulphonylureas (1.24, 0.93-1.65), and acarbose (4.21, 0.44-40.43) were not statistically associated with the risk of DR. With evidence from longitudinal studies only, insulin was found to have a higher risk of DR than other antidiabetic drugs (OR: 2.47, 95% CI: 2.04-2.99).

**Conclusion:**

Our results indicate that antidiabetic drugs are generally safe to prescribe regarding the risk of DR among people with type 2 diabetes. Further robust and large-scale trials investigating the effects of insulin, meglitinides, and acarbose on DR are warranted.

**Systematic review registration:**

https://www.crd.york.ac.uk/prospero/display_record.php?RecordID=332052, identifier CRD42022332052.

## Introduction

Type 2 diabetes accounts for 90% of the 537 million global cases of diabetes and is anticipated to reach 783 million by 2045 (1, 2). Along with the increasing number of individuals with type 2 diabetes, there is reason to be concerned over the long-term diabetes-related complications, attributed to the tissue-damaging effects of chronic hyperglycemia (3). The potentially alarming impact of these complications is manifested in the case of diabetic retinopathy (DR), a microvascular complication that impacts around one-third of people with type 2 diabetes throughout their lives, which is recognized as one of the five leading causes of blindness worldwide and has become a significant challenge to the healthcare systems (4, 5). The estimated cost of treating DR in people with type 2 diabetes in the UK was £51 million in 2010 and is predicted to rise to £87 million by 2035/36 (6).

There are nine classes of antidiabetic drugs for people with type 2 diabetes, including insulin, metformin, sulfonylureas, glucagon-like peptide-1 agonists (GLP-1 RA), sodium-glucose cotransporter-2 inhibitors (SGLT-2i), dipeptidyl peptidase-4 inhibitors (DPP-4i), thiazolidinediones, acarbose, and meglitinides. The effects of these drugs on glucose control have been well established, and some drugs’ additional benefits on related diabetic complications, such as GLP-1 RA, have been reported (7). However, their effects on DR remain uncertain, due to the variance in mechanisms of glucose control. For instance, GLP-1 RA operates by inhibiting insulin secretion and reducing glucagon release through enhancing the action of glucagon-like peptide-1 (8), whereas DPP-4i works through enhancing the action of incretin hormones (9), and SGLT-2i reduces renal glucose reabsorption (10). Previous systematic reviews and meta-analyses have focused on one drug class, one single drug, or only included randomized controlled trials (RCTs) or longitudinal observational studies, which limits the ability to properly inform clinical guidelines.

To provide reliable evidence to help inform clinical decisions about choices of glucose-lowering agents for people with type 2 diabetes, we conducted this umbrella review where we have systematically mapped and evaluated evidence from existing systematic reviews and meta-analyses of the effects of antidiabetic drugs on the risk of DR in people with type 2 diabetes.

## Methods

This umbrella review was conducted according to the pre-specified protocol registered in PROSPERO (CRD42022332052) and is reported following the Preferred Reporting Items for Overviews of Reviews (PRIOR) (11).

### Literature search

A literature search was conducted systematically using a predefined search strategy in Medline, Embase, and Cochrane Library for systematic reviews and meta-analyses that investigated the relationships between antidiabetic drugs and DR from inception till 17^th^ of May 2022. The selection of keywords for the study underwent a rigorous evaluation process, which overseen by clinicians, experts in ophthalmology, and epidemiologists. Keywords used in the search included the following domains: systematic review or meta-analysis, type 2 diabetes, antidiabetic drugs, and DR (full search strategies are provided in **Supplementary Material S1**).

### Study selection

Inclusion criteria were: 1) systematic reviews or meta-analyses of randomized controlled trials (RCTs) and/or cohort studies; 2) included people with type 2 diabetes; 3) intervention (exposure) was one of the following glucose-lowering drug classes, including insulin, metformin, sulphonylureas, GLP-1 RA, SGLT-2i, DPP-4i, thiazolidinediones, acarbose, and meglitinides; 4) Control (non-exposure) group should be placebo or any of other glucose-lowering drug classes; 5) the reported outcome was the presence of DR, accepting the definition in systematic reviews. Exposures, comparators, and outcomes were defined through a scoping search and after consultation with an expert panel (including ophthalmologists, diabetologists, and epidemiologists). There was no restriction on language or year of publication. The titles, abstracts, and full texts of the studies identified through the search were independently reviewed by two authors (LT and ZW). Disagreements were addressed by consensus and by seeking advice from a third person (JW).

### Data extraction

Two authors independently conducted data extraction (LT and ZW), and disagreements were addressed by discussing with the third person (JW). For each included systematic review or meta-analysis, we extracted the first author, year of publication, type of included studies, number of studies included, intervention, comparison, information on population, number of participants, characteristics of participants, outcome definition, search details, quality assessment tools, analysis methodology, evaluated results (effect size and 95% CIs), reporting heterogeneity, and findings (**Supplementary Table S1**). For any missing or unclear information, we accessed the included primary studies or contacted the authors of the included systematic reviews for further details.

### Quality assessment

AMSTAR-2 (A MeaSurement Tool to Assess Systematic Reviews) checklist was applied to evaluate the methodological quality for included reviews (**Supplementary Table S2**) (12). Two authors (LT and ZW) evaluated the quality of included reviews independently. Any disagreement was resolved by discussing with the third person (JW).

### Assessment of the degree of overlap

If two or more reviews focused on the same exposure and outcome and included similar primary studies, these studies were evaluated for their degree of overlapping relationships (13). Incorporating data from reviews with overlapping relationships could result in including primary studies more than once, causing bias in the estimates and results (14, 15). The degree of overlap was quantified by the measure of corrected covered area (CCA), which is classified as very high (CCA > 15%), high (CCA 11–15%), moderate (CCA 6–10%), and slight (CCA 0-5%) (**Supplementary Material S2**, **Supplementary Tables S3** to **S7**) (13). When there was overlap between two or more reviews, preference was given to the review with the highest quality and with the highest number of primary studies if there were two or more reviews with the same quality.

### Data analysis

Data were classified and analyzed according to study design (RCTs or cohort studies), drug classes, and comparison groups. We assessed and depicted the heterogeneity of each meta-analysis using *I* ^2^ statistics (16), where a value of *I* ^2^ over 50% indicated significant heterogeneity (17). Additionally, we estimated publication bias for comparison with at least 10 studies by using Egger’s statistical test, whereas P value less than 0.1 was considered statistically significant (18). For the comparison that included two or more studies, random-effects meta-analyses were used to update the preference review selected during overlapping assessment, by adding primary studies from other reviews. For comparison that included only one study, the original results were reported. We conducted a *post hoc* analysis to compare the classes of GLP-1 RA, because previous systematic reviews reported conflicting results and seven of the included systematic reviews and meta-analyses focused on GLP-1 RA or Semaglutide. All statistical analyses were performed in Review Manager 5.4 and R 4.12.

The GRADE (Grading of recommendations, Assessment, Development and Evaluation) was applied to evaluate and summarize the quality of evidence for each systematic review and meta-analysis included in the umbrella review, which grades the certainty of the evidence as high, mediate, low, or very low (**Supplementary Table S8**) (19).

## Results

### Literature search

**Figure 1:**
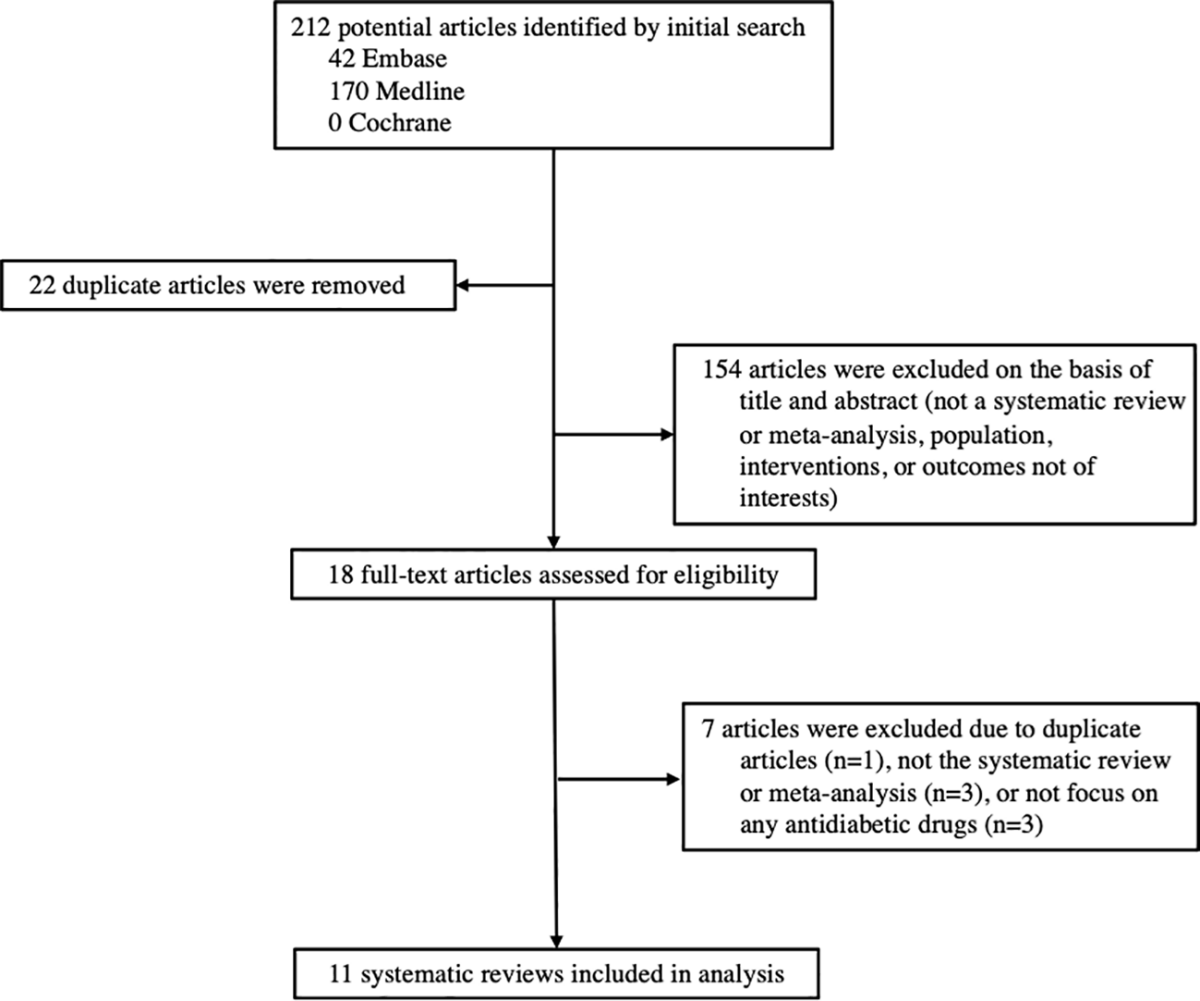
The flow chart of study selection.

Among the 212 records identified through our systematic search, 11 systematic reviews and meta-analyses investigated the associations between antidiabetic drugs and DR were included in this study, including 9 reviews of RCTs and 2 reviews of cohort studies (**Figure 1**). The characteristics of all included reviews are summarized and presented in **Table 1**. The list of excluded studies and the reasons for exclusion are provided in **Supplementary Table S9**.

**Table 1:** Characteristics of the systematic reviews and meta-analyses included for the associations between antidiabetic medication and diabetic retinopathy in people with type 2 diabetes.

Systematic reviews	Included Study types	No. Studies (related to DR)	Interventions/Exposures	Comparison	Definition of DR	Quality assessment tool	AMSTAR2
Wang, 2021 (20)	RCT	23	Semaglutide	Placebo or all classes of antidiabetic drugs	Incidence of DR	Cochrane	High
Tsapas, 2020 (21)	RCT	12	All classes antidiabetic drugs	Monotherapy of all classes’ antidiabetic drugs	DR	Cochrane	Low
Avgerinos, 2020 (22)	RCT	5	Semaglutide	Placebo or all classes of antidiabetic drugs.	DR	Cochrane	Low
Avgerinos, 2019 (23)	RCT	18	GLP-1	Placebo or all classes of antidiabetic drugs.	Incidence of DR	Cochrane	High
Tang, 2018 (24)	RCT	37	All classes antidiabetic drugs	Placebo, no treatment, or all classes of antidiabetic drugs	Reported DR: macular edema, vitreous hemorrhage, onset of diabetes-related blindness, and the need for treatment with an intravitreal agent or retinal photocoagulation	Cochrane	Moderate
Andreadis, 2018 (25)	RCT	12	Semaglutide	Placebo or all classes of antidiabetic drugs	Event of DR	Cochrane	High
Gargiulo, 2017 (26)	RCT	22	GLP-1	Placebo or all classes of antidiabetic drugs	DR: DR, diabetic retinal complications, and blurred vison related to DM	NA	Critically Low
Li, 2021 (27)	RCT	6	SGLT2	Placebo	DR: non-proliferative retinopathy, proliferative retinopathy, retinal oedema, haemorrhage or detachment	Cochrane	Low
Bethel, 2021 (28)	RCT	6	GLP-1	Placebo	DR	NA	Critically Low
Caparrotti, 2021 (29)	Cohort	1	GLP-1	Combination therapy of all classes’ antidiabetic drugs	DR	NA	High
Zhao, 2014 (30)	Cohort	7	Insulin	NA	DR	Newcastle-Ottawa Scale	Moderate

### Methodology quality

Out of the 11 included systematic reviews, four (36%) were rated as high quality (20, 23, 25, 29), two (18%) were moderate quality (24, 30), and five (45%) were low or critically low quality (21, 22, 26–28) (**Table 1** and **Supplementary Table S10**). The main limitations in the low or critically low quality studies included: 1) the risk of bias of the included primary studies was not assessed (n=2), 2) the list of the excluded studies with reasons was not provided (n=7), and 3) the statement of the study designs established before conducting review was not given (n=9).

### Overlapping and non-overlapping associations

CCA assessment revealed overlapping associations in 81% (n=17) comparisons within two or more systematic reviews. Two systematic reviews had non-overlapping associations. Detailed overlapping associations are provided in **Supplementary Tables S3** to **Table S7**.

### Summary findings

**Figure 2:**
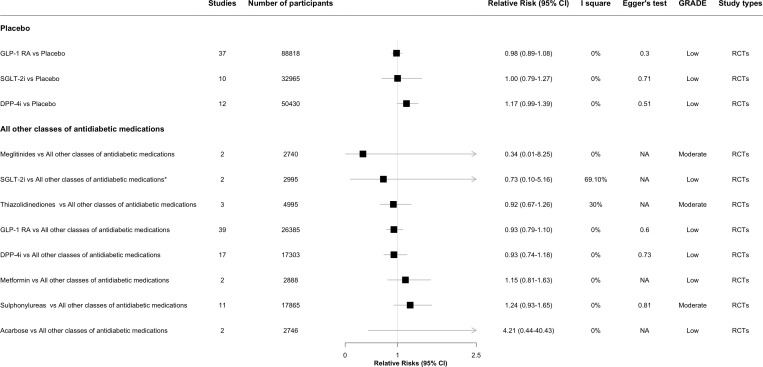
The effect of antidiabetic medications on diabetic retinopathy compared with placebo or all other classes of antidiabetic medications, by class. * This meta-analysis result was reported by included reviews. CI, confidence interval; ; GRADE, Grading of Recommendations, Assessment, Development and Evaluation; SGLT-2i, sodium-glucose cotransporter-2 inhibitors; GLP-1 RA, glucagon-like peptide-1 agonists; DPP-4i, dipeptidyl peptidase-4 inhibitors; NA, not available.

The results of meta-analyses regarding the associations between antidiabetic drugs and DR in comparison to placebo or all other classes of antidiabetic medications are presented in **Figures 2** through **3**, organized by drug class and individual drug. The **Supplementary Figures S1** and **S2** present the outcomes of the meta-analyses conducted to compare various drug classes.

**Figure 3:**
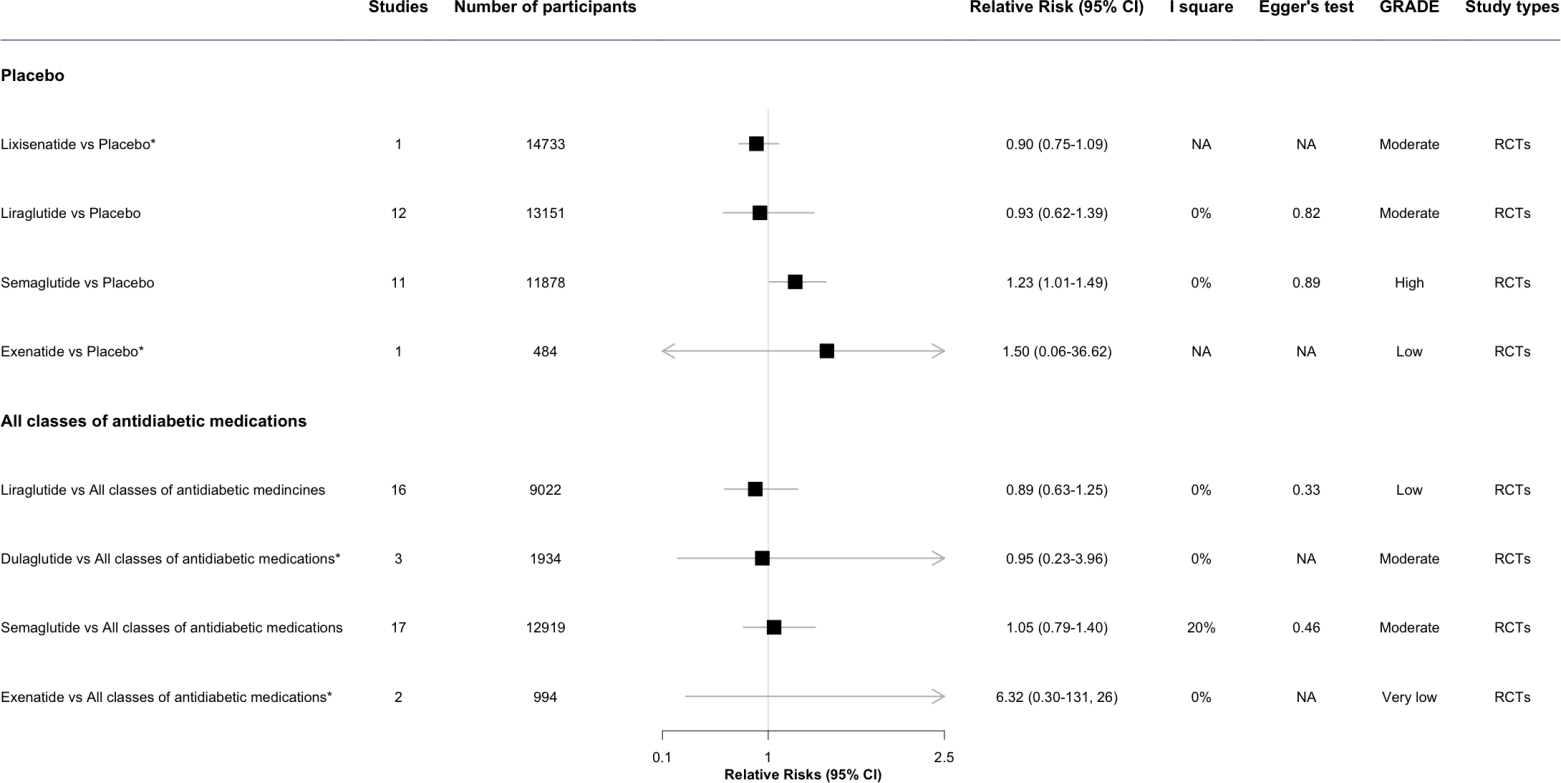
The forest plot of association between GLP-1 RA individual drugs and diabetic retinopathy compared with placebo and all other classes of antidiabetic medications. * This meta-analysis result was reported by included reviews. CI, confidence interval; GRADE, Grading of Recommendations, Assessment, Development and Evaluation; NA, not available.

### Antidiabetic drugs vs. placebo

In the overall analysis, the risk of DR development or progress was not evident for two antidiabetic drug classes: GLP-1 RA (RR: 0.98, 95%CI 0.89-1.08, *I^2^ = *0%) and SGLT-2i (1.00, 0.79-1.27, *I^2^ = *0%), when comparing each class of antidiabetic drug with placebo. DPP-4i was observed associated with an increased risk of developing DR (1.17, 0.99-1.39, *I^2^ = *0%), though the result was not statistically significant (**Figure 2**).

In the *post hoc* analysis among different individual GLP-1 RA, Semaglutide (1.23, 95% CI 1.01-1.49, *I* ^2^ = 0%) was associated with the increased risk of DR. For Lixisenatide (0.90, 0.75-1.09, *I* ^2^ = 0%) and Liraglutide (0.93, 0.62-1.39, *I* ^2^ = 0%), the pooled estimate showed no significant increased risk of DR (23). Evidence for exenatide was limited with only 1 event in 484 participants (**Figure 3**) (23).

### Antidiabetic drugs vs. all other antidiabetic drugs

**Figure 4:**
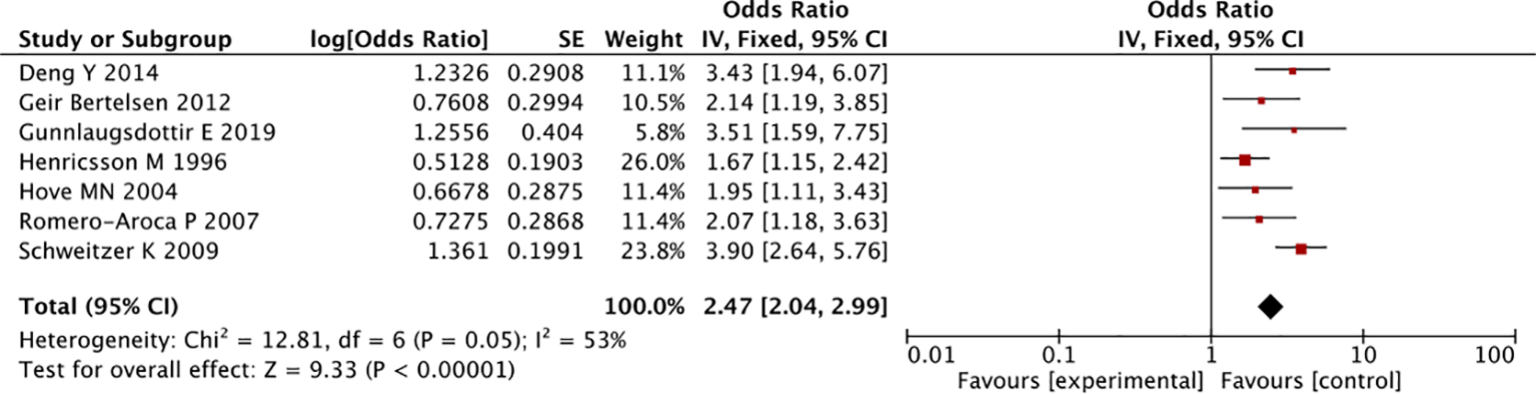
The forest plot of association between insulin and diabetic retinopathy.

In the overall analysis, there was no statistically significant association for any antidiabetic drug class, such as SGLT-2i (RR: 0.73, 95% CI 0.10-5.16, *I* ^2^ = 69.1%), thiazolidinediones (0.92, 0.67-1.26, *I* ^2^ = 30%), GLP-1 RA (0.93, 0.79-1.18, *I* ^2^ = 0%), DPP-4i (0.93, 0.74-1.18, *I* ^2^ = 0%), and metformin (1.15, 0.81-1.63, *I* ^2^ = 0%), compared with all other classes of antidiabetic drugs (**Figure 2**). Some signals were noted for sulphonylureas (1.24, 0.93-1.65, *I* ^2^ = 0%) and insulin treatment (OR: 2.47, 95%CI 2.04-2.99, *I* ^2^ = 53%), which were associated with an increased risk of developing DR, though the result of insulin was taken from longitudinal observational studies (**Figures 2**, **4**) (30).

In the *post hoc* analysis of individual drugs of GLP-1 RA, Liraglutide, Dulaglutide, and Semaglutide were not associated with the risk of DR. For Exenatide, no significantly increased risks of DR were observed in the pooled estimate, owing to insufficient evidence (**Figure 3**) (23).

In the comparison of each antidiabetic drug class side-by-side, sulphonylureas was associated with an increased risk of DR (RR: 1.41, 95% CI 1.00-2.00, *I* ^2^ = 0%), comparing with GLP-1 RA. There were no other antidiabetic drug class associated with a significantly higher risk of DR, which could be due to the limited evidence of these pairwise comparisons (**Supplementary Figure S1**).

**Supplementary Table S11** presents the meta-analysis findings for drug classes before and after updating the preferred review with additional missing studies noted in other reviews.

**Supplementary Table S12** provides all meta-analyses results in the umbrella review, including published results, individual studies results, updated pooled results, and GRADE-evaluated risk of bias. 15% (n=6) of the associations were graded as high or moderate, 51% (n=21) of associations were graded as low quality, and 34% (n=14) of associations were very low quality.

## Discussion

### Main findings

To the best of our understanding, this is the first umbrella review presenting a comprehensive update on the available evidence derived from RCTs and observational studies, focusing the association between nine classes of antidiabetic drugs and the risk of DR in individuals with type 2 diabetes. Our results indicate that antidiabetic drugs are generally safe to prescribe regarding the risk of DR among people with type 2 diabetes. However, signals were observed for DPP-4i (comparing with placebo), sulphonylureas (comparing with all classes of antidiabetic drugs), and insulin potentially associated with increasing the incidence of DR, though insulin association was based on evidence from observational studies (30). In the *post hoc* analysis, comparing with placebo, Semaglutide (an individual GLP-1 RA) was observed associated with an increased incidence of DR. This umbrella review offers insights for clinicians and policymakers, aiding them in making informed decisions about the selection of antidiabetic medications for individuals with diabetes and DR. Furthermore, our suggest a need for further robust and large-scale clinical trials to further investigate the effects of insulin, meglitinides, and acarbose on the development of DR.

Our results showed sulphonylureas might be related to an increased risk of DR, comparing with thiazolidinediones, SGLT-2i, GLP-1 RA, and DPP-4i. On the other hand, previous research has shown conflicting results. A retrospective chart review study reported that the odds ratio (OR) of DR for people receiving sulphonylureas was reduced to 0.45 (95% CI 0.28–0.71) compared to non-users (31). Results from a cohort study showed no significant association between sulphonylureas and the risk of DR when compared with metformin (hazard ratio: 1.02, 95%CI: 0.92-1.04) (32). Given the potential bias originating from observational studies, further research, preferably from randomized trial evidence is needed.

Our results suggested a potential correlation between DPP-4i and the increased risk of DR in people with type 2 diabetes, when compared with placebo. One possible hypothesis is that DPP-4i could potentially promote vascular leakage by elevating the concentration of Stromal Cell-Derived Factor 1 alpha, thereby facilitating the process of angiogenesis and vascular leakage (33). This could have an adverse effect on DR. However, the association identified in our study was mostly contributed by the Green et al. study (34), with a median follow-up duration of three years. A published cohort study reported the adverse effect of DPP-4i on DR may be limited to the initial phase of treatment, potentially attributable to the rapid glucose improvement (35). However, the mechanism of DPP-4i on DR remains unclear, and the existing experimental results have not yielded consistent results in terms of biomarker changes in DR after DPP-4 inhibitor initiation. Thus, future research and clinical trials are warranted.

Findings from a previous meta-analysis suggested that insulin treatment increased the risk of DR (30), which was similar to results from a hospital-based study (n=134), showing that insulin was associated with a higher risk of DR in people with type 2 diabetes, compared to people treated with other antidiabetic drugs (OR 2.4, 95%CI 0.9-6.6) (36). All this evidence was observational in nature which is prone to certain biases such as indication bias that may overestimate the effect of insulin of DR risk.

Our findings showed that SGLT-2i with no effect on DR, compared with placebo. However, this association was not statistically significant. This lack of association between SGLT-2i and DR may be attributed to the limited numbers of included studies and participants. A recent literature review stated SGLT-2i could slow the progression of DR (37), through the prevention of pericyte damage, which is a critical first step in the pathogenesis of DR (38). It has also been reported that SGLT-2i might delay the progression of DR by reducing oxidative stress, one of the major causes of DR that leads to retinal damage, eventually leading to DR (39).

In the *post hoc* analysis, our results indicated Semaglutide might be associated with the increased risk of DR in comparison with placebo. This results are consistent with previous published studies concerning the effect of Semaglutide on DR (40, 41). Although the exact mechanism by which of Semaglutide increase DR not established, a potential explanation is that it is due to the abrupt glycemic correction following the introduction of Semaglutide, reflected by a rapid decrease in rapid HbA_1c_ (42). Large and rapid reductions in blood glucose levels may lower intravascular osmotic pressure leading to an osmotic gradient between extracellular and intravascular areas. As water tends to flow from areas that have high osmotic to low osmotic pressure, this movement of water may have a greater effect on vessels that are low-pressure such as those in the eye. A breakdown of the blood-brain barrier and hypoxic environment (leading to VEGF upregulation) may provide the pathophysiological rationale for worsening of DR (43). Longer duration of type 2 diabetes, existing DR or other microvascular complications, higher baseline HbA_1c_, as well as the insulin treatment may also be significant predictor of this effect (44). Finally, the duration of trials might also provide an alternative framework for interpretation. It is therefore possible that this is not a direct effect of Semaglutide, but the net effect of several factors (including rapid decreases in glycated and ensuing osmotic changes in a setting of diminished counter-regulatory mechanisms, due to long standing type 2 diabetes) (45). However, when compared with all class of other antidiabetic drugs, Semaglutide was not associated with risk of DR.

Furthermore, with the exception of Semaglutide, our results showed that GLP-1 RA as a class were not significantly associated with DR, when compared with placebo and other drug classes. This finding is consistent with previous cardiovascular safety trials, where GLP-1 RA in were not associated with a higher risk of DR (46–49). Therefore, when Semaglutide is being considered as the next step in the treatment intensification in the setting of long-standing diabetes with pre-existing DR any detrimental effects may be counteracted by longer titration intervals between doses of Semaglutide, leading to a less steep decline in HbA_1c_.

After completing our study, we conducted an additional systematic literature search for cohort studies evaluating associations between antidiabetic drugs and DR in people with type 2 diabetes. Findings from recently published cohort studies were consistent with our results, such as the possible adverse impact of add-on DPP-4i treatment on DR (50), non-significant associations between GLP-1 RA and DR (51), as well as the potential association between combination therapy of SGLT-2i with metformin and the reduced risk of DR in individuals with type 2 diabetes (52).

### Strength and limitations

This umbrella review applied the stringent methodological umbrella review approach of the literature, by systematically synthesizing and appraising all available evidence from published meta-analyses. This allowed for a wide scope of the effect of antidiabetic drugs on the risk of DR, since analyses were undertaken both at the level of specific glucose-lowering class and for individual antidiabetic agents within each glucose-lowering class. Additionally, the quantitative comparisons between nine antidiabetic agents, provided in the present study, can enable health-care providers with a more reliable estimation of the effects on DR for a number of glucose-lowering agents.

On the other hand, the findings of the present study should be interpreted within the context of its limitations. This umbrella review was based on evidence from published systematic reviews and meta-analyses, and thus, potential limitations and shortcomings of the published literature, inherent to study design, might undermine the validity of the findings. The AMSTAR-2 and GRADE were applied to assess the methodology and evidence quality of included studies in this review. Importantly, the heterogeneity of baseline characteristics and insufficient data collection in primary studies may limit the interpretation. This is also the case when considering the different definitions of the DR-related outcomes of interest across previous systematic reviews, which prevented a uniform estimation of the term DR. We tried to overcome this by documenting all original definitions of DR were collected and reported in **Table 1**. As some of the baseline characteristics such as age, sex, ethnicity, the duration of type 2 diabetes, the dosage of drugs, or documentation of diabetic complications were missing in many studies, we were not able to explore their role as potential effect modifiers.

### Implications for clinical practice and public health

The high-level evidence from our study can provide useful information for clinicians when considering treatment intensification for achieving glycemic targets, especially in the setting of pre-existing DR or when risk factors for DR are present. This review also provides evidence for policy and guideline recommendations regarding the pharmacological management of type 2 diabetes and DR. For example, at the drug class level, GLP-1 RA were not significantly associated with the risk of developing DR, yet Semaglutide treatment might be correlated to a higher incidence of DR, comparing with placebo. Therefore, it might be plausible to assume that, when Semaglutide is considered the next step in treatment intensification, clinicians should verify that individuals with type 2 diabetes have been screened for the presence and severity of DR before treatment initiation. A holistic estimation of the risk-to-benefit ratio of the intervention should also be discussed and considered during the decision-making process. It might also be advisable that additional caution be applied in the setting of long-standing diabetes with pre-existing DR, which would allow for a less steep decline in HbA_1c_ and thus, a lesser risk of DR development or progression. Finally, a follow-up DR assessment within a shorter timeframe might be contemplated in the same setting.

### Implications for future research

Further exploration of the effect of antidiabetic drugs on DR outcomes is important since DR is one of the major causes of vision loss and blindness in adults, and the number of people at risk of DR is expected to increase. This study provides comprehensive evidence that antidiabetic drugs are generally safe to people at the risk of DR, while compared with placebo, Semaglutide may be associated with a higher incidence of DR. Results from the FOCUS RCT of the long-term effects of Semaglutide on DR in people with type 2 diabetes are eagerly anticipated (53). Furthermore, since primary studies included individuals both with and without baseline DR, it is not clear whether glucose-lowering drugs are associated with DR in people without evidence of DR at baseline.

## Conclusions

In conclusion, the findings of this umbrella review suggest that antidiabetic drugs are generally safe to prescribe regarding the risk of DR among people with type 2 diabetes.

However, this study reveals that at the individual drug level, Semaglutide is linked to an increased incidence of DR, and at the drug class level, DPP-4i, sulphonylureas, and insulin had a potential association with a slight higher incidence of DR. It is noteworthy that the evidence derived from observational studies for insulin, which may introduce indication bias, and there is an insufficiency of statistical power in investigations assessing the effects of sulphonylureas and DPP-4i. Further robust and large-scale trials investigating the effects of insulin, meglitinides, and acarbose on DR are warranted.

## Data availability statement

The original contributions presented in the study are included in the article/**Supplementary Material**. Further inquiries can be directed to the corresponding authors.

## Author contributions

LT: Conceptualization, Formal analysis, Investigation, Methodology, Writing – original draft. ZW: Data curation, Writing – review & editing. KO: Methodology, Writing – review & editing. KT: Writing – review & editing. AD: Supervision, Writing – review & editing. BS: Writing – review & editing. FC: Supervision, Writing – review & editing. CS: Supervision, Writing – review & editing. JW: Methodology, Supervision, Writing – review & editing. KN: Supervision, Writing – review & editing.
