# Supplementary material for: Associations of antidiabetic drugs with diabetic retinopathy in people with type 2 diabetes: an umbrella review and meta-analysis
Source: Front Endocrinol (Lausanne). 2024 Jan 3;14:1303238. doi: 10.3389/fendo.2023.1303238 (PMC10795175; doi:10.3389/fendo.2023.1303238)
Supplement: Supplementary file 1 [file DataSheet_1.docx]

Table of Contents

[Material S1: Search strategy 3](#_Toc144405618)

[1.1 Search strategy in Embase 3](#_Toc144405619)

[1.2 Search strategy in Medline 6](#_Toc144405620)

[1.3 Search strategy in Cochrane library 11](#_Toc144405621)

[Table S1: Data extraction form 14](#_Toc144405622)

[Table S2: AMSTAR2 form 15](#_Toc144405623)

[Material S2: The formula for calculating corrected covered area (CCA) 19](#_Toc144405624)

[Table S3: Citation matrices for reviews with overlapping associations about the risk of GLP-1 agonists in DR 20](#_Toc144405625)

[Table S4: Citation matrices for reviews with overlapping associations about the risk of Semaglutide in DR 22](#_Toc144405626)

[Table S5: Citation matrices for reviews with overlapping associations about the risk of other individual GLP-1 agonists in DR 24](#_Toc144405627)

[Table S6: Citation matrices for reviews with overlapping associations about the risk of DPP4 inhibitors in DR 27](#_Toc144405628)

[Table S7: Citation matrices for reviews with overlapping associations about the risk of SGLT2 inhibitors in DR 29](#_Toc144405629)

[Table S8: GRADE of quality evidence for antidiabetic medications on DR 30](#_Toc144405630)

[Table S9: Excluded systematic reviews 32](#_Toc144405631)

[Figure S1: The forest plot of the comparison within different drug classes 33](#_Toc144405632)

[Table S10: AMSTAR2 items for systematic reviews and meta-analyses included in the umbrella review 36](#_Toc144405633)

[Table S11: Table of meta-analysis results before and after updating 37](#_Toc144405634)

[Table S12: Quantitative synthesis, and bias assessment of 41 meta-analyses of antidiabetic drugs on DR 38](#_Toc144405635)

[Figure S3:Forest plot of SGLT-2 inhibitors vs Placebo 40](#_Toc144405636)

[Figure S4:Forest plot of GLP-1 agonists vs Placebo 41](#_Toc144405637)

[Figure S5: Forest plot of DPP4 inhibitors vs Placebo 42](#_Toc144405638)

[Figure S6: Forest plot of Meglitinides vs All other classes of antidiabetic drugs 43](#_Toc144405639)

[Figure S7: Forest plot of TZD vs All other classes of antidiabetic drugs 44](#_Toc144405640)

[Figure S8: Forest plot of GLP-1 agonists vs All other classes of antidiabetic drugs 45](#_Toc144405641)

[Figure S9: Forest plot of DPP4 inhibitors vs All other classes of antidiabetic drugs 46](#_Toc144405642)

[Figure S10: Forest plot of SU vs All other classes of antidiabetic drugs 47](#_Toc144405643)

[Figure S11: Forest plot of Acarbose vs All other classes of antidiabetic drugs 48](#_Toc144405644)

[Figure S12: Forest plot of Metformin vs All other classes of antidiabetic drugs 49](#_Toc144405645)

[Figure S13: Forest plot of Semaglutide vs Placebo 50](#_Toc144405646)

[Figure S14: Forest plot of Semaglutide vs All other classes of antidiabetic drugs 51](#_Toc144405647)

[Figure S15: Forest plot of Semaglutide vs DPP-4 inhibitors 52](#_Toc144405648)

[Figure S16: Forest plot of Semaglutide vs other GLP-1 agonists 53](#_Toc144405649)

[Figure S17: Forest plot of Liraglutide vs Placebo 54](#_Toc144405650)

[Figure S18: Forest plot of Liraglutide vs All other classes of antidiabetic drugs 55](#_Toc144405651)

[Figure S24: Funnel plot of GLP-1 agonists vs Placebo 61](#_Toc144405652)

[Figure S25: Funnel plot of DPP4 inhibitors vs Placebo 62](#_Toc144405653)

[Figure S26: Funnel plot of GLP-1 agonists vs All other classes of antidiabetic drugs 63](#_Toc144405654)

[Figure S27: Funnel plot of DPP4 inhibitors vs All other classes of antidiabetic drugs 64](#_Toc144405655)

[Figure S28: Funnel plot of SU vs All other classes of antidiabetic drugs 65](#_Toc144405656)

[Figure S30: Funnel plot of Semaglutide vs All other classes of antidiabetic drugs 67](#_Toc144405657)

[Figure S31: Funnel plot of Liraglutide vs Placebo 68](#_Toc144405658)

[Figure S32: Funnel plot of Liraglutide vs All other classes of antidiabetic drugs 69](#_Toc144405659)

[Figure S33: Funnel plot of GLP-1 agonists vs TZD 70](#_Toc144405660)

[Figure S34: Funnel plot of GLP-1 agonists vs Insulin 71](#_Toc144405661)

[Figure S35: Funnel plot of GLP-1 agonists vs DPP4 72](#_Toc144405662)

[Figure S36: Funnel plot of GLP-1 agonists vs SU 73](#_Toc144405663)

# **Material S1: Search strategy**

## **1.1 Search strategy in Embase**

1 exp Diabetes Mellitus, Type 2/

2 exp Diabetic Retinopath*/ or Diabetic retinopath*/

3 (diabetic retinopath* or diabetic eye disease).tw.

4 proliferative diabetic retinopathy.tw.

5 diabetic macular edema.tw.

6 diabetic macular oedema.tw.

7 diabetic maculopathy.tw.

8 retinal disorders.tw.

9 vision loss.tw.

10 Insulin/ or Insulins/ or Insulin Aspart/ or Insulin Detemir/ or Insulin Glargine/ or Insulin, Isophane/ or Insulin Lispro/ or Insulin, Regular, Human/ or Insulin, Lente/ or Isophane Insulin, Human/ or Insulin, Ultralente/ or exp Insulin, Long Acting/

11 ((insulin adj1 aspart) or (insulin adj1 detemir) or (insulin adj1 glargine) or (insulin adj1 isophane) or (insulin adj1 lispro) or ((long acting or longer acting or intermediate acting) adj insulin*) or (insulin adj1 lente) or (insulin adj1 ultralente)).tw.

12 (insulin adj1 degludec).tw.

13 (insulin adj1 zinc).tw.

14 (insulin adj1 glulisine).tw.

15 Biphasic Insulins/

16 (novorapid or fiasp).mp.

17 Biguanides/ or biguanides.tw. or Metformin/ or metformin.tw.

18 alpha-Glucosidases/ or alpha glucosidase inhibitor*.tw.

19 Acarbose/ or acarbose.tw.

20 miglitol.tw.

21 voglibose.tw.

22 Amylin Receptor Agonists/ or (amylin adj1 (analogue* or derivative*)).tw. or pramlintide.tw.

23 meglitinide*.tw.

24 mitiglinide.tw.

25 Nateglinide/ or nateglinide.tw.

26 repaglinide.tw.

27 glitazone*.tw.

28 Pioglitazone/ or pioglitazone.tw.

29 rivoglitazone.tw.

30 Rosiglitazone/ or rosiglitazone.tw.

31 lobeglitazone.tw.

32 Receptors, Glucagon/ag or Glucagon-Like Peptide-1 Receptor/ or Glucagon-Like Peptide 1/ag

33 (glucagon-like peptide 1 receptor inhibitor* or glucagon-like peptide 1 receptor agonist* or glucagon-like peptide 1 inhibitor* or glucagon-like peptide 1 agonist* or GLP-1 receptor inhibitor* or GLP-1 receptor agonist* or GLP-1 inhibitor* or GLP-1 agonist*).tw.

34 albiglutide.tw.

35 dulaglutide.tw.

36 (exenatide or exendin 4).tw.

37 Liraglutide/ or liraglutide.tw.

38 lixisenatide.tw.

39 semaglutide.tw.

40 taspoglutide.tw.

41 Sodium-Glucose Transporter 2 Inhibitors/ or (sodium glucose transporter 2 inhibitor* or sodium glucose transporter ii inhibitor* or SGLT 2 inhibitor* or (sodium glucose cotransporter adj3 inhibitor*) or (sodium glucose co transporter adj3 inhibitor*)).tw.

42 Canagliflozin/ or canagliflozin.tw.

43 dapagliflozin.tw.

44 empagliflozin.tw.

45 ertugliflozin.tw.

46 ipragliflozin.tw.

47 luseogliflozin.tw.

48 remogliflozin etabonate.tw.

49 sotagliflozin.tw.

50 tofogliflozin.tw.

51 Sulfonylurea Compounds/ or (sulphonylurea or sulphonylureas).tw.

52 Acetohexamide/ or acetohexamide.tw.

53 Carbutamide/ or carbutamide.tw.

54 Chlorpropamide/ or chlorpropamide.tw.

55 glibenclamide.tw.

56 glibornuride.tw.

57 Gliclazide/ or gliclazide.tw.

58 glimepiride.tw.

59 Glipizide/ or glipizide.tw.

60 gliquidone.tw.

61 glisoxepide.tw.

62 Glyburide/ or glyburide.tw.

63 glyclopyramide.tw.

64 glycopyramide.tw.

65 glycyclamide.tw.

66 metahexamide.tw.

67 Tolazamide/ or tolazamide.tw.

68 Tolbutamide/ or tolbutamide.tw.

69 tolcyclamide.tw.

70 Dipeptidyl-Peptidase IV Inhibitors/ or (dipeptidyl-peptidase IV Inhibitor* or dipeptidyl-peptidase 4 Inhibitor* or ((DPP4 or DPP 4 or DPP IV) adj inhibitor*)).tw.

71 alogliptin.tw.

72 anagliptin.tw.

73 dutogliptin.tw.

74 evogliptin.tw.

75 gemigliptin.tw.

76 gosogliptin.tw.

77 Linagliptin/ or linagliptin.tw.

78 omarigliptin.tw.

79 saxagliptin.tw.

80 septagliptin.tw.

81 Sitagliptin Phosphate/ or (sitagliptin or Sitagliptin Phosphate).tw.

82 teneligliptin.tw.

83 trelagliptin.tw.

84 Vildagliptin/ or vildagliptin.tw.

85 Hypoglycemic Agents/

86 or/10-85

87 exp review/

88 (literature adj3 review$).ti,ab.

89 exp meta analysis/

90 exp "Systematic Review"/

91 87 or 88 or 89 or 90

92 (medline or medlars or embase or pubmed or cinahl or amed or psychlit or psyclit or psychinfo or psycinfo or scisearch or cochrane).ti,ab.

93 RETRACTED ARTICLE/

94 92 or 93

95 91 and 94

96 (systematic$ adj2 (review$ or overview)).ti,ab.

97 (meta?anal$ or meta anal$ or meta-anal$ or metaanal$ or metanal$).ti,ab.

98 95 or 96 or 97

99 2 or 3 or 4 or 5 or 6 or 7 or 8 or 9

100 1 and 86 and 99

101 98 and 100

## **1.2 Search strategy in Medline**

1 Diabetes Mellitus.mp. or exp Diabetes Mellitus/

2 (Diabetic Retinopath* or Diabetic retinopath*).mp. [mp=title, abstract, original title, name of substance word, subject heading word, floating sub-heading word, keyword heading word, organism supplementary concept word, protocol supplementary concept word, rare disease supplementary concept word, unique identifier, synonyms]

3 (diabetic retinopath* or diabetic eye disease).mp. [mp=title, abstract, original title, name of substance word, subject heading word, floating sub-heading word, keyword heading word, organism supplementary concept word, protocol supplementary concept word, rare disease supplementary concept word, unique identifier, synonyms]

4 exp Diabetic Retinopathy/ or proliferative diabetic retinopathy.mp.

5 exp Visual Acuity/ or exp Macular Edema/ or exp Diabetic Retinopathy/ or diabetic macular edema.mp.

6 exp Macular Edema/ or exp Diabetic Retinopathy/ or diabetic macular oedema.mp. or exp Visual Acuity/

7 exp Visual Acuity/ or exp Diabetic Retinopathy/ or diabetic maculopathy.mp. or exp Macular Edema/

8 exp Retinal Diseases/ or exp Vision Disorders/ or retinal disorders.mp.

9 exp Diabetic Retinopathy/ or exp Macular Degeneration/ or vision loss.mp. or exp Blindness/ or exp Vision Disorders/

10 exp Insulin, Isophane/ or exp Isophane Insulin, Human/ or insulin.mp. or exp Insulin Detemir/ or exp Insulin Aspart/ or exp Insulin, Long-Acting/ or exp Insulin Glargine/ or exp Insulin/ or exp Insulin, Ultralente/ or exp Insulin Lispro/ or exp Insulin, Regular, Human/ or exp Insulin, Lente/

11 ((insulin adj1 aspart) or (insulin adj1 detemir) or (insulin adj1 glargine) or (insulin adj1 isophane) or (insulin adj1 lispro) or ((long acting or longer acting or intermediate acting) adj insulin*) or (insulin adj1 lente) or (insulin adj1 ultralente)).mp. [mp=title, abstract, original title, name of substance word, subject heading word, floating sub-heading word, keyword heading word, organism supplementary concept word, protocol supplementary concept word, rare disease supplementary concept word, unique identifier, synonyms]

12 (insulin adj1 degludec).mp. [mp=title, abstract, original title, name of substance word, subject heading word, floating sub-heading word, keyword heading word, organism supplementary concept word, protocol supplementary concept word, rare disease supplementary concept word, unique identifier, synonyms]

13 (insulin adj1 zinc).mp. [mp=title, abstract, original title, name of substance word, subject heading word, floating sub-heading word, keyword heading word, organism supplementary concept word, protocol supplementary concept word, rare disease supplementary concept word, unique identifier, synonyms]

14 (insulin adj1 glulisine).mp. [mp=title, abstract, original title, name of substance word, subject heading word, floating sub-heading word, keyword heading word, organism supplementary concept word, protocol supplementary concept word, rare disease supplementary concept word, unique identifier, synonyms]

15 Biphasic Insulins.mp. or exp Insulin/ or exp Biphasic Insulins/

16 (novorapid or fiasp).mp. [mp=title, abstract, original title, name of substance word, subject heading word, floating sub-heading word, keyword heading word, organism supplementary concept word, protocol supplementary concept word, rare disease supplementary concept word, unique identifier, synonyms]

17 Biguanide.mp. or Biguanides/

18 biguanides.mp. or exp Biguanides/

19 Metformin.mp. or exp Metformin/

20 alpha-Glucosidases.mp. or exp alpha-Glucosidases/

21 exp Acarbose/ or exp alpha-Glucosidases/ or alpha glucosidase inhibitor*.mp.

22 acarbose.mp. or exp Acarbose/

23 miglitol.mp.

24 voglibose.mp.

25 Amylin Receptor Agonists.mp. or exp Amylin Receptor Agonists/

26 pramlintide.mp.

27 (amylin adj1 (analogue* or derivative*)).mp. [mp=title, abstract, original title, name of substance word, subject heading word, floating sub-heading word, keyword heading word, organism supplementary concept word, protocol supplementary concept word, rare disease supplementary concept word, unique identifier, synonyms]

28 25 or 26 or 27

29 meglitinide*.mp.

30 mitiglinide.mp.

31 Nateglinide.mp. or exp Nateglinide/

32 repaglinide.mp.

33 glitazone*.mp.

34 Pioglitazone.mp. or exp Pioglitazone/

35 exp Thiazolidinediones/ or rivoglitazone.mp.

36 Rosiglitazone.mp. or exp Rosiglitazone/

37 exp Thiazolidinediones/ or lobeglitazone.mp.

38 Receptors, Glucagon/ag

39 Glucagon-Like Peptide-1 Receptor.mp. or exp Glucagon-Like Peptide-1 Receptor

40 Glucagon-Like Peptide 1/ag

41 38 or 39 or 40

42 exp Hypoglycemic Agents/ or exp Diabetes Mellitus, Type 2/ or exp Glucagon-Like Peptide 1/ or glucagon-like peptide 1 receptor inhibitor*.mp. or exp Receptors, Glucagon/ or exp Glucagon-Like Peptide-1 Receptor/

43 GLP-1 agonist*.mp.

44 GLP-1 inhibitor*.mp.

45 GLP-1 receptor agonist*.mp.

46 exp Receptors, Glucagon/ or exp Glucagon-Like Peptide-1 Receptor/ or exp Glucagon-Like Peptides/ or albiglutide.mp. or exp Glucagon-Like Peptide 1/

47 dulaglutide.mp.

48 exenatide.mp. or exp Exenatide/

49 exendin 4.mp.

50 Liraglutide.mp. or exp Liraglutide/

51 lixisenatide.mp.

52 semaglutide.mp.

53 taspoglutide.mp.

54 Sodium-Glucose Transporter 2 Inhibitors/ or (sodium glucose transporter 2 inhibitor* or sodium glucose transporter ii inhibitor* or SGLT 2 inhibitor* or (sodium glucose cotransporter adj3 inhibitor*) or (sodium glucose co transporter adj3 inhibitor*)).mp. [mp=title, abstract, original title, name of substance word, subject heading word, floating sub-heading word, keyword heading word, organism supplementary concept word, protocol supplementary concept word, rare disease supplementary concept word, unique identifier, synonyms]

55 Canagliflozin.mp. or exp Canagliflozin/

56 dapagliflozin.mp.

57 empagliflozin.mp.

58 ertugliflozin.mp.

59 ipragliflozin.mp.

60 luseogliflozin.mp.

61 remogliflozin etabonate.mp.

62 sotagliflozin.mp. or exp Sodium-Glucose Transporter 2/ or exp Sodium-Glucose Transporter 2 Inhibitors/ or exp Diabetes Mellitus, Type 2/

63 Sulfonylurea Compounds.mp. or exp Sulfonylurea Compounds/

64 sulphonylurea.mp. or exp Sulfonylurea Compounds/

65 sulphonylureas.mp. or exp Sulfonylurea Compounds/

66 63 or 64 or 65

67 Acetohexamide.mp. or exp Acetohexamide/

68 Carbutamide.mp. or exp Carbutamide/

69 chlorpropamide.mp. or exp Chlorpropamide/

70 glibenclamide.mp. or exp Glyburide/

71 glibornuride.mp.

72 Gliclazide.mp. or exp Gliclazide/

73 glimepiride.mp.

74 Glipizide.mp. or exp Glipizide/

75 gliquidone.mp.

76 glisoxepide.mp.

77 Glyburide.mp. or Glyburide/

78 glyclopyramide.mp.

79 glycopyramide.mp.

80 glycyclamide.mp.

81 metahexamide.mp.

82 Tolazamide.mp. or exp Tolazamide/

83 Tolbutamide.mp. or exp Tolbutamide/

84 tolcyclamide.mp.

85 exp Dipeptidyl Peptidase 4/ or exp Dipeptidyl-Peptidase IV Inhibitors/ or exp "Dipeptidyl-Peptidases and Tripeptidyl-Peptidases"/

86 exp Dipeptidyl-Peptidase IV Inhibitors/ or alogliptin.mp.

87 exp Dipeptidyl Peptidase 4/ or anagliptin.mp.

88 exp Dipeptidyl-Peptidase IV Inhibitors/ or dutogliptin.mp.

89 exp Dipeptidyl-Peptidase IV Inhibitors/ or evogliptin.mp.

90 gemigliptin.mp.

91 gosogliptin.mp.

92 Linagliptin.mp. or exp Linagliptin/

93 omarigliptin.mp.

94 saxagliptin.mp.

95 Sitagliptin Phosphate.mp. or exp Sitagliptin Phosphate/

96 (sitagliptin or Sitagliptin Phosphate).mp. [mp=title, abstract, original title, name of substance word, subject heading word, floating sub-heading word, keyword heading word, organism supplementary concept word, protocol supplementary concept word, rare disease supplementary concept word, unique identifier, synonyms]

97 95 or 96

98 itagliptin Phosphate/ or (sitagliptin or Sitagliptin Phosphate).mp. [mp=title, abstract, original title, name of substance word, subject heading word, floating sub-heading word, keyword heading word, organism supplementary concept word, protocol supplementary concept word, rare disease supplementary concept word, unique identifier, synonyms]

99 teneligliptin.mp.

100 trelagliptin.mp.

101 Vildagliptin.mp. or exp Vildagliptin/

102 Hypoglycemic Agents.mp. or exp Hypoglycemic Agents/

103 or/10-102

104 or/2-9

105 1 and 103 and 104

106 review.pt.

107 (medline or medlars or embase or pubmed or cochrane).tw,sh.

108 (scisearch or psychinfo or psycinfo).tw,sh.

109 (psychlit or psyclit).tw,sh.

110 cinahl.tw,sh.

111 ((hand adj2 search$) or (manual$ adj2 search$)).tw,sh.

112 (electronic database$ or bibliographic database$ or computeri?ed database$ or online database$).tw,sh.

113 (pooling or pooled or mantel haenszel).tw,sh.

114 (peto or dersimonian or der simonian or fixed effect).tw,sh.

115 (retraction of publication or retracted publication).pt.

116 or/107-115

117 106 and 116

118 meta-analysis.pt.

119 meta-analysis.sh.

120 (meta-analys$ or meta analys$ or metaanalys$).tw,sh.

121 (systematic$ adj5 review$).tw,sh.

122 (systematic$ adj5 overview$).tw,sh.

123 (quantitativ$ adj5 review$).tw,sh.

124 (quantitativ$ adj5 overview$).tw,sh.

125 (quantitativ$ adj5 synthesis$).tw,sh.

126 (methodologic$ adj5 review$).tw,sh.

127 (methodologic$ adj5 overview$).tw,sh.

128 (integrative research review$ or research integration).tw.

129 or/119-128

130 118 or 129

131 117 or 130

132 105 and 131

133 type 2 diabetes.mp. or exp Diabetes Mellitus, Type 2/

134 132 and 133

## **1.3 Search strategy in Cochrane library**

#1 MeSH descriptor: [Diabetic Retinopathy] explode all trees

#2 (proliferative diabetic retinopathy):ti,ab,kw

#3 (diabetic macular edema):ti,ab,kw

#4 (diabetic macular oedema):ti,ab,kw

#5 (diabetic maculopathy):ti,ab,kw

#6 (retinal disorders):ti,ab,kw

#7 (vision loss):ti,ab,kw

#8 #1 OR #2 OR #3 OR #4 OR #5 OR #6 OR #7

#9 (Insulin) or (Insulins) or (Insulin Aspart) or (Insulin Detemir) or (Insulin Glargine) or (Insulin, Isophane) or (Insulin Lispro) or (Insulin, Regular, Human) or (Insulin, Lente) or (Isophane Insulin, Human) or (Insulin, Ultralente) or (exp Insulin, Long Acting)

#10 ((insulin NEAR/3 aspart) or (insulin NEAR/3 detemir) or (insulin NEAR/3 glargine) or (insulin NEAR/3 isophane) or (insulin NEAR/3 lispro) or ((long acting or longer acting or intermediate acting) NEAR/3 insulin*) or (insulin NEAR/3 lente) or (insulin NEAR/3 ultralente)):ti,ab,kw

#11 (insulin NEAR/3 degludec):ti,ab,kw

#12 (insulin NEAR/3 zinc):ti,ab,kw

#13 (insulin NEAR/3 glulisine):ti,ab,kw

#14 Biphasic Insulins

#15 (novorapid or fiasp):ti,ab,kw

#16 ((Biguanides or biguanides) or (Metformin or metformin)):ti,ab,kw

#17 (Alpha-Glucosidases or alpha glucosidase inhibitor*):ti,ab,kw

#18 (Acarbose or acarbose):ti,ab,kw

#19 (miglitol):ti,ab,kw

#20 (voglibose):ti,ab,kw

#21 (Amylin Receptor Agonists or (amylin NEAR/3 (analogue* or derivative*)) or pramlintide):ti,ab,kw

#22 (meglitinide*):ti,ab,kw

#23 (mitiglinide):ti,ab,kw

#24 (Nateglinide or nateglinide):ti,ab,kw

#25 (repaglinide):ti,ab,kw

#26 (glitazone*):ti,ab,kw

#27 (Pioglitazone or pioglitazone):ti,ab,kw

#28 (rivoglitazone):ti,ab,kw

#29 (Rosiglitazone or rosiglitazone):ti,ab,kw

#30 (lobeglitazone):ti,ab,kw

#31 (Receptors, Glucagon/ag or Glucagon-Like Peptide-1 Receptor or Glucagon-Like Peptide 1/ag):ti,ab,kw

#32 (glucagon-like peptide 1 receptor inhibitor* or glucagon-like peptide 1 receptor agonist* or glucagon-like peptide 1 inhibitor* or glucagon-like peptide 1 agonist* or GLP-1 receptor inhibitor* or GLP-1 receptor agonist* or GLP-1 inhibitor* or GLP-1 agonist*):ti,ab,kw

#33 (albiglutide):ti,ab,kw

#34 (dulaglutide):ti,ab,kw

#35 (exenatide or exendin 4):ti,ab,kw

#36 (Liraglutide or liraglutide):ti,ab,kw

#37 (lixisenatide):ti,ab,kw

#38 (semaglutide):ti,ab,kw

#39 (taspoglutide):ti,ab,kw

#40 (Sodium-Glucose Transporter 2 Inhibitors or (sodium glucose transporter 2 inhibitor* or sodium glucose transporter ii inhibitor* or SGLT 2 inhibitor* or (sodium glucose cotransporter adj3 inhibitor*) or (sodium glucose co transporter adj3 inhibitor*))):ti,ab,kw

#41 (Canagliflozin or canagliflozin):ti,ab,kw

#42 (dapagliflozin):ti,ab,kw

#43 (empagliflozin):ti,ab,kw

#44 (ertugliflozin):ti,ab,kw

#45 (ipragliflozin):ti,ab,kw

#46 (luseogliflozin):ti,ab,kw

#47 (remogliflozin etabonate):ti,ab,kw

#48 (sotagliflozin):ti,ab,kw

#49 (tofogliflozin):ti,ab,kw

#50 (Sulfonylurea Compounds or (sulphonylurea or sulphonylureas)):ti,ab,kw

#51 (Acetohexamide or acetohexamide):ti,ab,kw

#52 (Carbutamide or carbutamide):ti,ab,kw

#53 (Chlorpropamide or chlorpropamide):ti,ab,kw

#54 (glibenclamide):ti,ab,kw

#55 (glibornuride):ti,ab,kw

#56 (Gliclazide or gliclazide):ti,ab,kw

#57 (glimepirid):ti,ab,kw

#58 (Glipizide or glipizide):ti,ab,kw

#59 (gliquidone):ti,ab,kw

#60 (glisoxepide):ti,ab,kw

#61 (Glyburide or glyburide):ti,ab,kw

#62 (glyclopyramide):ti,ab,kw

#63 (glycopyramide):ti,ab,kw

#64 (glycyclamide):ti,ab,kw

#65 (metahexamide):ti,ab,kw

#66 (Tolazamide or tolazamide):ti,ab,kw

#67 (Tolbutamide or tolbutamide):ti,ab,kw

#68 (tolcyclamide):ti,ab,kw

#69 (Dipeptidyl-Peptidase IV Inhibitors or (dipeptidyl-peptidase IV Inhibitor* or dipeptidyl-peptidase 4 Inhibitor* or ((DPP4 or DPP 4 or DPP IV) NEAR/3 inhibitor*))):ti,ab,kw

#70 (alogliptin):ti,ab,kw

#71 (anagliptin):ti,ab,kw

#72 (dutogliptin):ti,ab,kw

#73 (evogliptin):ti,ab,kw

#74 (gemigliptin):ti,ab,kw

#75 (gosogliptin):ti,ab,kw

#76 (Linagliptin or linagliptin):ti,ab,kw

#77 (omarigliptin):ti,ab,kw

#78 (saxagliptin):ti,ab,kw

#79 (septagliptin):ti,ab,kw

#80 (Sitagliptin Phosphate or (sitagliptin or Sitagliptin Phosphate)):ti,ab,kw

#81 (teneligliptin):ti,ab,kw

#82 (trelagliptin):ti,ab,kw

#83 (Vildagliptin or vildagliptin):ti,ab,kw

#84 Hypoglycemic Agents

#85 #9 OR #10 OR #11 OR #12 OR #13 OR #14 OR #15 OR #16 OR #17 OR #18 OR #19 OR #20 OR #21 OR #22 OR #23 OR #24 OR #25 OR #26 OR #27 OR #28 OR #29 OR #30 OR #31 OR #32 OR #33 OR #34 OR #35 OR #36 OR #37 OR #38 OR #39 OR #40 OR #41 OR #42 OR #43 OR #44 OR #45 OR #46 OR #47 OR #48 OR #49 OR #50 OR #51 OR #52 OR #53 OR #54 OR #55 OR #56 OR #57 OR #58 OR #59 OR #60 OR #61 OR #62 OR #63 OR #64 OR #65 OR #66 OR #67 OR #68 OR #69 OR #70 OR #71 OR #72 OR #73 OR #74 OR #75 OR #76 OR #77 OR #78 OR #79 OR #80 OR #81 OR #82 OR #83 OR #84

#86 Diabetes Mellitus, Type 2

#87 #8 AND #85 in Cochrane Reviews

#88 #8 AND #85 AND #86 in Cochrane Reviews

# **Table S1: Data extraction form**

| **Study details** | First author |
| --- | --- |
|  | Year of publication |
|  | Type of studies |
|  | Number of studies |
|  | Characteristics |
|  | Total number |
|  | Interventions/Exposure |
|  | Control / Comparisons |
|  | Outcomes definitions |
| **Search details** | Sources searched |
|  | Range (years) of included studies |
| **Quality appraisal** | Appraisal instruments |
|  | Methods of analysis |
| **Analysis** | Outcome assessed (effect size and 95% CIs) |
|  | Heterogeneity (95% CI) |
| **Results** | Findings |

# **Table S2: AMSTAR2 form**

| **AMSTAR** **2**  **1.** **Did** **the** **research** **questions** **and** **inclusion** **criteria** **for** **the** **review** **include** **the** **components** **of** | | |
| --- | --- | --- |
| **PICO?**  For Yes:  Population Intervention  Comparator Outcome  group  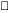  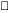  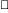  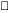 | \| Optional (recommended)  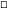 Timeframe for follow-up  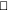 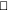  Yes No \| \| --- \| | |
| **2.** **Did** **the** **report** **of** **the** **review** **contain** **an** **explicit** **statement** **that** **the** **review** **methods** **were** **established** **prior** **to** **the** **conduct** **of** **the** **review** **and** **did** **the** **report** **justify** **any** **significant** **deviations** **from** **the** **protocol?** | | |
| \| For Partial Yes:  For Yes:  As for partial yes, plus the protocol should be registered and should also have specified:  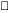 a meta-analysis/synthesis plan, if appropriate, *and*  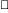 a plan for investigating causes of heterogeneity  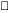 justification for any deviations from the protocol  The authors state that they had a written  protocol or guide that included ALL the  following:  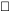 review question(s)  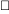 a search strategy  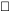 inclusion/exclusion criteria  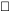 a risk of bias assessment \| \| --- \| | | \| 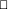 Yes  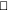 Partial Yes  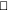 No \| \| --- \| |
| **3.** **Did** **the** **review** **authors** **explain** **their** **selection** **of** **the** **study** **designs** **for** **inclusion** **in** **the** **review?**   \| For Yes, the review should satisfy ONE of the following:  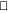 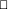  Yes No  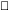 *Explanationfor* including only RCTs  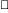 OR *Explanationfor* including only NRSI  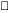 OR *Explanationfor* including both RCTs and NRSI \| \| --- \|   **4.** **Did** **the** **review** **authors** **use** **a** **comprehensive** **literature** **search** **strategy?**   \| For Yes, should also have (all the  For Partial Yes (all the following):  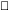 searched at least 2 databases (relevant to research question)  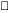 provided key word and/or search strategy  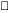 justified publication restrictions (eg, language)  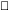 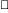 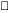  Yes Partial Yes No  following):  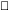 searched the reference lists/bibliographies of included studies  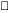 searched trial/study registries  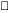 included/consulted content experts in the field  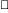 where relevant, searched for grey literature  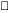 conducted search within 24 months of completion of the review \| \| --- \|   **5.** **Did** **the** **review** **authors** **perform** **study** **selection** **in** **duplicate?**   \| For Yes, either ONE of the following:  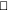 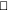  Yes No  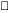 at least two reviewers independently agreed on selection of eligible studies and achieved consensus on which studies to include  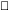 OR two reviewers selected a sample of eligible studies and achieved good agreement (at least 80 per cent), with the remainder selected by one reviewer \| \| --- \| | | |
| **6.** **Did** **the** **review** **authors** **perform** **data** **extraction** **in** **duplicate?** | | |
| For Yes, either ONE of the following:  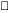 at least two reviewers achieved consensus on which data to extract  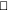 Yes | | |

| \| from included studies 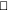 No  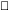 OR two reviewers extracted data from a sample of eligible studies and achieved good agreement (at least 80 per cent), with the remainder extracted by one reviewer \| \| --- \|   **7.** **Did** **the** **review** **authors** **provide** **a** **list** **of** **excluded** **studies** **and** **justify** **the** **exclusions?**   \| For Yes, must also have:  For Partial Yes:  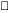 Justified the exclusion from  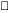 provided a list of all  potentially relevant studies that were read in full text form but excluded from the review  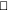 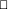 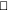  Yes Partial Yes No  the review of each  potentially relevant study \| \| --- \|   **8.** **Did** **the** **review** **authors** **describe** **the** **included** **studies** **in** **adequate** **detail?**   \| For Yes, should also have ALL the  For Partial Yes (ALL the following):  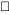 described populations  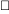 described interventions  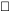 described comparators  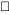 described outcomes  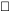 described research designs  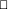 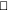 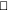  Yes Partial Yes No  following:  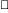 described population in detail  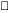 described intervention and comparator in detail (including doses where relevant)  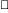 described study’s setting  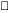 timeframe for follow-up \| \| --- \|   **9.** **Did** **the** **review** **authors** **use** **a** **satisfactory** **technique** **for** **assessing** **the** **risk** **of** **bias** **(RoB)** **in** | | |
| --- | --- | --- | --- | --- | --- |
| **individual** **studies** **that** **were** **included** **in** **the** **review?**   \| **RCTs** \|  \| \| --- \| --- \| \| For Partial Yes, must have assessed \| For Yes, must also have assessed \| \| RoB from \| RoB from: \| \| 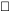 unconcealed allocation, *and* \| 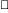 allocation sequence that was \| \| 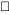 lack of blinding of patients and assessors when assessing outcomes (unnecessary for objective outcomes such as all cause mortality) \| not truly random, *and* \| \| 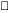 selection of the reported \| \| result from among multiple \| \| measurements or analyses of \| \| a specified outcome \| \| **NRSI** \|  \| \| For Partial Yes, must have assessed \| For Yes, must also have assessed \| \| RoB: \| RoB: \| \| 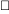 from confounding, *and* \| 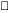 methods used to ascertain \| \| 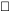 from selection bias \| exposures and outcomes,  *and*  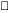 selection of the reported result from among multiple measurements or analyses of a specified outcome \| | | \| Yes  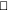 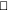  Partial Yes \| \| --- \| \| 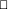 No \| \| 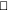 Includes only \| \| NRSI \| \| Yes  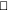 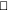  Partial Yes \| \| 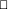 No \| \| 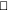 Includes only \| \| RCTs \| \|  \| |
| **10.** **Did** **the** **review** **authors** **report** **on** **the** **sources** **of** **funding** **for** **the** **studies** **included** **in** **the** **review?**   \| For Yes  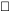 Must have reported on the sources of funding for individual studies included  Yes No  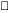 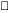  in the review. Note: Reporting that the reviewers looked for this information  but it was not reported by study authors also qualifies \| \| --- \|   **11.** **If** **meta-analysis** **was** **performed** **did** **the** **review** **authors** **use** **appropriate** **methods** **for** **statistical** **combination** **of** **results?** | | |
| **RCTs**  For Yes:  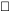 The authors justified combining the data in a meta-analysis  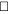 AND they used an appropriate weighted technique to combine study results and adjusted for heterogeneity if present | 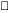 Yes  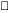 No  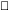 No meta-analysis | |

| \| 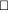 AND investigated the causes of any heterogeneity \| \| conducted \| \| --- \| --- \| --- \| \| **For** **NRSI**  For Yes:  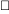 The authors justified combining the data in a meta-analysis  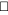 AND they used an appropriate weighted technique to combine study results, adjusting for heterogeneity if present  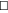 AND they statistically combined effect estimates from NRSI that were adjusted for confounding, rather than combining raw data, or justified combining raw data when adjusted effect \| \| 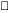 Yes  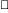 No  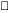 No meta-analysis conducted \| \| 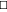 \| estimates were not available  AND they reported separate summary estimates for RCTs and \| \| NRSI separately when both were included in the review \| \|   **12.** **If** **meta-analysis** **was** **performed,** **did** **the** **review** **authors** **assess** **the** **potential** **impact** **of** **RoB** **in** **individual** **studies** **on** **the** **results** **of** **the** **meta-analysis** **or** **other** **evidence** **synthesis?**   \| For Yes:  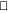 Yes  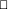 No  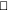 No meta-analysis conducted  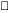 included only low risk of bias RCTs  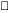 OR, if the pooled estimate was based on RCTs and/or NRSI at variable RoB, the authors performed analyses to investigate possible impact of RoB on summary estimates of effect \| \| --- \|   **13.** **Did** **the** **review** **authors** **account** **for** **RoB** **in** **individual** **studies** **when** **interpreting/discussing** | |
| --- | --- | --- | --- | --- | --- | --- | --- | --- | --- | --- | --- | --- |
| **the** **results** **of** **the** **review?**   \| For Yes:  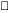 included only low risk of bias RCTs  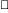 OR, if RCTs with moderate or high RoB, or NRSI were included the review provided a discussion of the likely impact of RoB on the results \| \| --- \| | \| 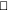 Yes  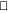 No \| \| --- \| |
| **14.** **Did** **the** **review** **authors** **provide** **a** **satisfactory** **explanation** **for,** **and** **discussion** **of,** **any** **heterogeneity** **observed** **in** **the** **results** **of** **the** **review?**   \| For Yes:  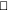  Yes No  There was no significant heterogeneity in the results  OR if heterogeneity was present the authors performed an investigation of sources of any heterogeneity in the results and discussed the impact of this on the results of the review \| \| --- \| | |
| **15.** **If** **they** **performed** **quantitative** **synthesis** **did** **the** **review** **authors** **carry** **out** **an** **adequate** **investigation** **of** **publication** **bias** **(small** **study** **bias)** **and** **discuss** **its** **likely** **impact** **on** **the** **results** | |
| **of** **the** **review?**   \| For Yes:  performed graphical or statistical tests for publication bias and discussed the likelihood and magnitude of impact of publication bias \| \| --- \| | \| Yes  No  No meta-analysis  conducted \| \| --- \| |
| **16.** **Did** **the** **review** **authors** **report** **any** **potential** **sources** **of** **conflict** **of** **interest,** **including** **any** **funding** **they** **received** **for** **conducting** **the** **review?** | |
| \| For Yes: \|  \|  \|  \| \| --- \| --- \| --- \| --- \| \|  \| The authors reported no competing interests OR \|  \| Yes \| \|  \| The authors described their funding sources and how they \|  \| No \| \|  \| managed potential conflicts of interest \|  \|  \| | |

# **Material S2: The formula for calculating corrected covered area (CCA)**

# **Table S3: Citation matrices for reviews with overlapping associations about the risk of GLP-1 agonists in DR**

| **Systematic Review ID** | Avgerinos 2019* | Tang 2018 | Bethel 2021 | Gargiulo 2017 | Wang 2022 | Tsapas 2020 | Avgerinos 2019* | Gargiulo 2017 | Tang 2018 | Tsapas 2020 | Andreadis 2018 | Wang 2022 | Tang 2018 | Andreadis 2018 | Avgerinos 2019* | Gargiulo 2017 | Tang 2018 | Tsapas 2020 | Avgerinos 2019 | Gargiulo 2017 | Tang 2018* | Gargiulo 2017 |
| --- | --- | --- | --- | --- | --- | --- | --- | --- | --- | --- | --- | --- | --- | --- | --- | --- | --- | --- | --- | --- | --- | --- |
| **Overlapping association** | Comparing with placebo | | | | | | Comparing with all classes of antidiabetic drugs | | | | | | Comparing with SU | | | | Comparing with Insulin | | | | Comparing with TZD | |
| **Primary Study** |  |  |  |  |  |  |  |  |  |  |  |  |  |  |  |  |  |  |  |  |  |  |
| Ahren 2013 |  | x |  |  |  |  |  |  |  |  |  |  |  |  |  |  |  |  |  |  |  |  |
| Ahren 2014 |  | x |  |  |  |  |  | x | x |  |  |  | x |  |  | x |  |  |  |  |  |  |
| Ahren 2017 |  |  |  |  |  |  | x |  |  |  | x |  |  |  |  |  |  |  |  |  |  |  |
| Araki 2015 |  |  |  |  |  |  | x | x | x |  |  |  |  |  |  |  | x |  | x | x |  |  |
| Aroda 2017 |  |  |  |  |  |  | x |  |  |  | x |  |  | x | x |  |  |  |  |  |  |  |
| Aroda 2019 |  |  |  |  | x |  |  |  |  | x |  |  |  |  |  |  |  | x |  |  |  |  |
| Bergenstal 2009 |  |  |  |  |  |  | x |  |  |  |  |  |  |  |  |  |  |  | x |  |  |  |
| Bethel 2020 |  |  | x |  |  |  |  |  |  |  |  |  |  |  |  |  |  |  |  |  |  |  |
| Billings 2018 |  |  |  |  |  |  |  |  |  | x |  |  |  |  |  |  |  | x |  |  |  |  |
| Blonde 2015 |  |  |  |  |  |  |  |  |  | x |  |  |  |  |  |  |  | x |  |  |  |  |
| Blonde 2020 |  |  |  |  |  | x |  |  |  |  |  |  |  |  |  |  |  |  |  |  |  |  |
| Davies 2016 | x |  |  |  |  |  |  |  |  |  |  |  |  |  |  |  |  |  |  |  |  |  |
| Davies 2021 |  |  |  |  | x |  |  |  |  |  |  |  |  |  |  |  |  |  |  |  |  |  |
| Garber 2011 |  |  |  |  |  |  | x | x |  |  |  |  |  |  | x | x |  |  |  |  |  |  |
| Gerstein 2019 |  |  | x |  |  |  |  |  |  |  |  |  |  |  |  |  |  |  |  |  |  |  |
| Gough 2014 |  |  |  |  |  |  | x |  |  |  |  |  |  |  |  |  |  |  |  |  |  |  |
| Hernandez 2018 |  |  | x |  |  |  |  |  |  |  |  |  |  |  |  |  |  |  |  |  |  |  |
| Holman 2017 | x | x | x |  |  |  |  |  |  |  |  |  |  |  |  |  |  |  |  |  |  |  |
| Home PD 2015 |  |  |  | x |  |  |  | x | x |  |  |  |  |  |  |  |  |  |  |  | x | x |
| Husain 2019 |  |  | x |  | x |  |  |  |  |  |  |  |  |  |  |  |  |  |  |  |  |  |
| Ji 2021 |  |  |  |  |  |  |  |  |  |  |  | x |  |  |  |  |  |  |  |  |  |  |
| Kaku 2011 |  |  |  |  |  |  | x | x | x |  |  |  | x |  | x | x |  |  |  |  |  |  |
| Kaku 2018 |  |  |  |  |  |  | x |  |  |  | x |  |  |  |  |  |  |  |  |  |  |  |
| Leiter 2014 |  |  |  |  |  |  |  |  | x |  |  |  |  |  |  |  |  |  |  |  |  |  |
| Lingvay 2018 |  |  |  |  | x |  |  |  |  |  |  | x |  |  |  |  |  |  |  |  |  |  |
| Lingvay 2019 |  |  |  |  |  |  |  |  |  |  |  | x |  |  |  |  |  |  |  |  |  |  |
| Marre 2009 | x |  |  |  |  |  |  |  |  |  |  |  |  |  |  |  |  |  |  |  |  |  |
| Marso 2016a | x | x | x | x |  | x |  |  |  |  |  |  |  |  |  |  |  |  |  |  |  |  |
| Marso 2016b | x | x | x | x |  |  |  |  |  |  |  |  |  |  |  |  |  |  |  |  |  |  |
| Mathieu 2014 |  |  |  |  |  |  | x |  |  |  |  |  |  |  |  |  |  |  | x |  |  |  |
| Mosenzon 2019 |  |  |  |  | x |  |  |  |  |  |  |  |  |  |  |  |  |  |  |  |  |  |
| Nauck 2013 | x |  |  |  |  |  | x |  |  |  |  |  |  |  | x |  |  |  |  |  |  |  |
| Nauck 2014 |  |  |  | x |  |  |  |  |  |  | x |  |  |  |  |  |  |  |  |  |  |  |
| Nauck 2016 | x | x |  | x |  |  |  |  |  |  |  |  |  |  |  |  |  |  |  |  |  |  |
| NCT00839527 2016 |  | x |  |  |  |  |  |  |  |  |  |  |  |  |  |  |  |  |  |  |  |  |
| NCT00849056 2016 |  | x |  |  |  |  |  |  |  |  |  |  |  |  |  |  |  |  |  |  |  |  |
| NCT02461589 | x |  |  |  |  |  |  |  |  |  |  |  |  |  |  |  |  |  |  |  |  |  |
| NCT01644500 2015 |  |  |  |  |  |  | x | x | x |  |  |  | x |  | x | x |  |  |  |  |  |  |
| NCT00960661 2015 |  |  |  |  |  |  | x | x | x |  |  |  |  |  |  |  | x |  | x | x |  |  |
| NCT01648582 2015 |  |  |  |  |  |  | x | x |  |  |  |  |  |  |  |  |  |  | x | x |  |  |
| Pfeffer 2015 |  | x |  | x |  |  |  |  |  |  |  |  |  |  |  |  |  |  |  |  |  |  |
| Pinget 2013 | x | x |  | x |  |  |  |  |  |  |  |  |  |  |  |  |  |  |  |  |  |  |
| Pratley 2011 |  |  |  |  |  |  | x | x | x | x |  |  |  |  |  |  |  |  |  |  |  |  |
| Pratley 2018 |  |  |  |  |  |  |  |  |  |  | x | x |  |  |  |  |  |  |  |  |  |  |
| Pratley 2019 |  |  |  |  | x | x |  |  |  | x |  | x |  |  |  |  |  |  |  |  |  |  |
| Pieber 2019 |  |  |  |  |  |  |  |  |  |  |  | x |  |  |  |  |  |  |  |  |  |  |
| Reusch 2014 |  |  |  | x |  |  |  |  |  |  |  |  |  |  |  |  |  |  |  |  |  |  |
| Rodbard 2018 | x |  |  |  | x |  |  |  |  |  |  |  |  |  |  |  |  |  |  |  |  |  |
| Rodbard 2019 |  |  |  |  |  |  |  |  |  |  |  | x |  |  |  |  |  |  |  |  |  |  |
| Rosenstock 2014 |  | x |  |  |  | x |  | x | x |  |  |  |  |  |  |  | x |  |  | x |  |  |
| Russell Jones 2009 | x |  |  |  |  |  | x |  |  | x |  |  |  |  |  |  |  | x | x |  |  |  |
| Rosenstock 2019 |  |  |  |  |  |  |  |  |  |  |  | x |  |  |  |  |  |  |  |  |  |  |
| Seino 2008 | x |  |  |  |  |  |  |  |  |  |  |  |  |  |  |  |  |  |  |  |  |  |
| Seino 2011 | x | x |  |  |  |  |  |  |  |  |  |  |  |  |  |  |  |  |  |  |  |  |
| Seino 2016 | x | x |  | x |  |  |  |  |  |  |  |  |  |  |  |  |  |  |  |  |  |  |
| Seino 2018 |  |  |  |  |  |  | x |  |  |  | x |  |  |  |  |  |  |  |  |  |  |  |
| Weissman 2014 |  |  |  |  |  |  |  | x | x |  |  |  |  |  |  |  | x |  |  | x |  |  |
| Xu 2013 |  |  |  |  |  |  |  | x |  |  |  |  |  |  |  |  |  |  |  | x |  | x |
| Yabe 2020 |  |  |  |  |  |  |  |  |  |  |  | x |  |  |  |  |  |  |  |  |  |  |
| Yamada 2019 |  |  |  |  |  |  |  |  |  |  |  | x |  |  |  |  |  |  |  |  |  |  |
| Yang 2011 |  |  |  |  |  |  | x |  |  |  |  |  |  |  | x |  |  |  |  |  |  |  |
| Zang 2016 |  |  |  |  |  |  | x |  |  |  |  |  |  |  |  |  |  |  |  |  |  |  |
| Zinman 2009 | x |  |  |  |  | x |  |  |  |  |  |  |  |  |  |  |  |  |  |  |  |  |
| Zinman 2019 |  |  |  |  | x |  |  |  |  |  |  |  |  |  |  |  |  |  |  |  |  |  |
| Zinman 2019 |  |  |  |  | x | x |  |  |  |  |  |  |  |  |  |  |  |  |  |  |  |  |
| Total (No. publications per review) | 15 | 13 | 7 | 9 | 9 | 6 | 18 | 12 | 10 | 6 | 6 | 10 | 3 | 1 | 6 | 4 | 4 | 5 | 5 | 6 | 1 | 2 |
| Grand Total (N) | 59 | | | | | | 62 | | | | | | 14 | | | | 20 | | | | 3 | |
| Rows (r) | 37 | | | | | | 39 | | | | | | 7 | | | | 12 | | | | 2 | |
| Columns (c) | 6 | | | | | | 6 | | | | | | 4 | | | | 4 | | | | 2 | |
| **CCA (%)** | 11.35 | | | | | | 15.38 | | | | | | 28.57 | | | | 22.22 | | | | 50 | |

**Corrected covered area (CCA) (%) = N-r / rc-r:**

**Where N = number of included primary studies (sum of checked boxes), r =number of rows (primary studies), c = number of columns (number of reviews).**

***: The selected systematic review and meta-analysis.**

# **Table S4: Citation matrices for reviews with overlapping associations about the risk of Semaglutide in DR**

| **Systematic Review ID** | Wang 2022* | Avgerinos 2020 | Avgerinos 2019 | Andreadis 2018 | Tsapas 2020 | Wang 2022* | Avgerinos 2020 | Avgerinos 2019 | Andreadis 2018 | Tsapas 2020 | Gargiulo 2017 | Tang 2018 | Andreadis 2018* | Tsapas 2020 | Avgerinos 2019 | Gargiulo 2017 | Tang 2018 | Wang 2022 | Andreadis 2018* | Wang 2022 | Tsapas 2020 |
| --- | --- | --- | --- | --- | --- | --- | --- | --- | --- | --- | --- | --- | --- | --- | --- | --- | --- | --- | --- | --- | --- |
| **Overlapping association** | Comparing with placebo | | | | | Comparing with all classes of antidiabetic drugs | | | | | | | Comparing with DPP4 inhibitors | | | | | | Comparing with other GLP-1 individual drugs | | |
| **Primary Study** |  |  |  |  |  |  |  |  |  |  |  |  |  |  |  |  |  |  |  |  |  |
| Ahren 2014 |  |  |  |  |  |  |  |  |  |  | x | x |  |  |  | x | x |  |  |  |  |
| Ahren 2017 |  |  |  |  |  | x |  | x | x |  |  |  | x |  | x |  |  |  |  |  |  |
| Aroda 2017 |  |  |  |  |  | x |  | x | x |  |  |  |  |  |  |  |  |  |  |  |  |
| Aroda 2019 | x | x |  |  |  |  |  |  |  |  |  |  |  |  |  |  |  |  |  |  |  |
| Capehorn 2019 |  |  |  |  |  | x |  |  |  |  |  |  |  |  |  |  |  |  |  |  |  |
| Davies 2021 | x | x |  |  |  |  |  |  |  |  |  |  |  |  |  |  |  |  |  |  |  |
| Husain 2019 | x | x |  |  | x |  |  |  |  |  |  |  |  |  |  |  |  |  |  |  |  |
| Ji 2021 |  |  |  |  |  | x |  |  |  |  |  |  |  |  |  |  |  | x |  |  |  |
| Kaku 2018 |  |  |  |  |  | x |  | x | x |  |  |  |  |  | x |  |  |  |  |  |  |
| Lingvay 2018 | x |  |  |  |  | x |  |  |  |  |  |  |  |  |  |  |  |  |  | x |  |
| Lingvay 2019 |  |  |  |  |  | x |  |  |  |  |  |  |  |  |  |  |  |  |  |  |  |
| Marso 2016 | x |  | x | x | x |  |  |  |  |  |  |  |  |  |  |  |  |  |  |  |  |
| Mosenzon 2019 | x | x |  |  | x |  |  |  |  |  |  |  |  |  |  |  |  |  |  |  |  |
| Nauck 2016 |  |  |  | x |  |  |  |  | x |  |  |  |  |  |  |  |  |  | x |  |  |
| Pieber 2019 |  |  |  |  |  | x | x |  |  |  |  |  |  |  |  |  |  | x |  |  |  |
| Pratley 2018 |  |  |  |  |  | x |  |  |  |  |  |  |  |  |  |  |  |  | x | x |  |
| Pratley 2019 | x | x |  |  | x | x | x |  |  |  |  |  |  |  |  |  |  |  |  | x | x |
| Rodbard 2018 | x |  | x |  | x |  |  |  |  |  |  |  |  |  |  |  |  |  |  |  |  |
| Rodbard 2019 |  |  |  |  |  | x | x |  |  |  |  |  |  |  |  |  |  |  |  |  |  |
| Rosenstock 2019 |  |  |  |  |  | x | x |  |  | x |  |  |  | x |  |  |  | x |  |  |  |
| Seino 2018 |  |  |  |  |  | x |  | x | x | x |  |  | x | x | x |  |  |  |  |  |  |
| Yabe 2020 |  |  |  |  |  | x |  |  |  |  |  |  |  |  |  |  |  |  |  | x |  |
| Yamada 2019 |  |  |  |  |  | x |  |  |  |  |  |  |  |  |  |  |  |  |  | x |  |
| Zinman 2019 | x | x |  |  | x |  |  |  |  |  |  |  |  |  |  |  |  |  |  |  |  |
| Zinman 2019 | x |  |  |  | x |  |  |  |  |  |  |  |  |  |  |  |  |  |  |  |  |
| Total (No of publications per review) | 11 | 6 | 2 | 2 | 8 | 15 | 4 | 4 | 5 | 2 | 1 | 1 | 2 | 2 | 3 | 1 | 1 | 3 | 2 | 5 | 1 |
| Grand Total (N) | 29 | | | | | 32 | | | | | | | 12 | | | | | | 8 | | |
| Rows (r) | 11 | | | | | 17 | | | | | | | 7 | | | | | | 6 | | |
| Columns (c) | 5 | | | | | 7 | | | | | | | 6 | | | | | | 3 | | |
| **CCA (%)** | 40.91 | | | | | 14.71 | | | | | | | 14.29 | | | | | | 16.67 | | |

**Corrected covered area (CCA) (%) = N-r / rc-r:**

**Where N = number of included primary studies (sum of checked boxes), r =number of rows (primary studies), c = number of columns (number of reviews).**

***: The selected systematic review and meta-analysis.**

# **Table S5: Citation matrices for reviews with overlapping associations about the risk of other individual GLP-1 agonists in DR**

| **Systematic review ID** | Avgerinos 2019* | Tsapas 2020 | Tang 2018 | Bethel 2021 | Gargiulo 2017 | Wang 2022 | Avgerinos 2019* | Tsapas 2020 | Andreadis 2018 | Gargiulo 2017 | Tang 2018 | Wang 2022 |
| --- | --- | --- | --- | --- | --- | --- | --- | --- | --- | --- | --- | --- |
| **Overlapping associations** | Liraglutide comparing with placebo | | | | | | Liraglutide comparing with all classes of antidiabetic drugs | | | | | |
| **Primary Study** |  |  |  |  |  |  |  |  |  |  |  |  |
| Ahren 2013 |  |  |  |  |  |  |  |  |  |  |  |  |
| Araki 2015 |  |  |  |  |  |  |  |  |  |  |  |  |
| Aroda 2019 |  |  |  |  |  |  |  | x |  |  |  |  |
| Bergenstal 2009 |  |  |  |  |  |  |  |  |  |  |  |  |
| Billings 2018 |  |  |  |  |  |  |  | x |  |  |  |  |
| Blonde 2015 |  |  |  |  |  |  |  |  |  |  |  |  |
| Capehorn 2019 |  |  |  |  |  |  |  | x |  |  |  |  |
| Davies 2016 | x |  |  |  |  |  |  |  |  |  |  |  |
| Diamant 2014 |  |  |  |  |  |  |  |  |  |  |  |  |
| Garber 2011 |  |  |  |  |  |  | x |  |  | x |  |  |
| Gough 2015 |  |  |  |  |  |  | x |  |  |  |  |  |
| Kaku 2011 |  |  |  |  |  |  | x |  |  | x | x |  |
| Lingvay 2018 |  |  |  |  |  |  |  |  |  |  |  | x |
| Marre 2009 | x |  |  | x | x |  |  |  |  |  |  |  |
| Mathieu 2014 |  |  |  |  |  |  | x |  |  |  |  |  |
| Marso 2016a | x | x | x |  |  |  |  |  |  |  |  |  |
| Nauck 2016 | x |  |  |  |  |  |  |  | x |  |  |  |
| Nauck 2013 | x |  |  |  |  |  | x |  |  |  |  |  |
| NCT00960661 2015 |  |  |  |  |  |  |  |  |  |  |  |  |
| NCT02461589 | x |  |  |  |  |  |  |  |  |  |  |  |
| NCT01644500 2015 |  |  |  |  |  |  |  |  |  |  |  |  |
| NCT01648582 2015 |  |  |  |  |  |  |  |  |  |  |  |  |
| Pfeffer 2015 |  |  |  |  |  |  |  |  |  |  |  |  |
| Pinget 2013 |  |  |  |  |  |  |  |  |  |  |  |  |
| Pratley 2011 |  |  |  |  |  |  | x |  |  |  |  |  |
| Pratley 2019 |  | x |  |  |  | x | x | x |  | x | x |  |
| Rosenstock 2014 |  |  |  |  |  |  |  |  |  |  |  |  |
| Russell Jones 2009 | x | x |  |  |  |  | x | x |  |  |  |  |
| Seino 2008 | x |  |  |  |  |  |  |  |  |  |  |  |
| Seino 2011 | x |  | x |  |  |  |  |  |  |  |  |  |
| Seino 2016 | x |  | x |  | x |  |  |  |  |  |  |  |
| Xu 2013 |  |  |  |  |  |  |  |  |  |  |  |  |
| Yamada 2019 |  |  |  |  |  |  |  |  |  |  |  | x |
| Yang 2011 |  |  |  |  |  |  | x |  |  |  |  |  |
| Zang 2016 |  |  |  |  |  |  | x |  |  |  |  |  |
| Zinman 2009 | x | x |  |  |  |  |  |  |  |  |  |  |
| Total (No of publications per review) | 11 | 4 | 3 | 1 | 2 | 1 | 10 | 5 | 1 | 3 | 2 | 3 |
| Grand Total (N) | 22 | | | | | | 24 | | | | | |
| Rows (r) | 12 | | | | | | 16 | | | | | |
| Columns (c) | 6 | | | | | | 6 | | | | | |
| **CCA (%)** | 16.67 | | | | | | 10 | | | | | |

**Corrected covered area (CCA) (%) = N-r / rc-r:**

**Where N = number of included primary studies (sum of checked boxes), r =number of rows (primary studies), c = number of columns (number of reviews).**

***: The selected systematic review and meta-analysis.**

# **Table S6: Citation matrices for reviews with overlapping associations about the risk of DPP4 inhibitors in DR**

| **Systematic review ID** | Tsapas 2020 | Tang 2018* | Tsapas 2020 | Tang 2018* |
| --- | --- | --- | --- | --- |
| **Overlapping associations** | Comparing with placebo | | Comparing with SU | |
| **Primary Study** |  |  |  |  |
| Ahren 2014 | x | x | x | x |
| Araki 2019 | x |  |  |  |
| Arjona Ferreira 2013 |  |  | x | x |
| Bamett 2013 |  | x |  |  |
| Chen 2018 | x |  |  |  |
| Green 2015 | x | x |  |  |
| Jadzinsky 2009 | x |  |  |  |
| Ownes NCT00602472 |  | x |  |  |
| Rosenstock 2019 | x |  | x |  |
| Rosenstock 2019 | x |  |  |  |
| Scirica 2013 | x | x |  |  |
| White 2013 | x | x |  |  |
| YKI-JÄRVINEN 2013 |  | x |  |  |
| Total (No. publications per review) | 9 | 7 | 3 | 2 |
| Grand Total (N) | 16 | | 5 | |
| Rows (r) | 12 | | 3 | |
| Columns (c) | 2 | | 2 | |
| **CCA (%)** | 33.33 | | 66.67 | |

**Corrected covered area (CCA) (%) = N-r / rc-r:**

**Where N = number of included primary studies (sum of checked boxes), r =number of rows (primary studies), c = number of columns (number of reviews).**

***: The selected systematic review and meta-analysis.**

# **Table S7: Citation matrices for reviews with overlapping associations about the risk of SGLT2 inhibitors in DR**

| **Systematic review ID** | Tsapas 2020 | Tang 2018* | Li 2021 |
| --- | --- | --- | --- |
| **Overlapping associations** | Comparing with placebo | | |
| **Primary Study** |  |  |  |
| Haring 2013 | x |  |  |
| Inagaki 2016 |  |  | x |
| Kashiwagi 2015 |  |  | x |
| Kovacs 2014 | x | x |  |
| Neal 2017 |  |  | x |
| Perkovic 2019 |  |  | x |
| Wanner 2016 |  | x |  |
| Yang 2016 |  | x | x |
| Zinman 2015 | x |  | x |
| Total (No. publications per review) | 3 | 3 | 6 |
| Grand Total (N) | 12 | | |
| Rows (r) | 9 | | |
| Columns (c) | 3 | | |
| **CCA (%)** | 12.5 | | |

**Corrected covered area (CCA) (%) = N-r / rc-r:**

**Where N = number of included primary studies (sum of checked boxes), r =number of rows (primary studies), c = number of columns (number of reviews).**

***: The selected systematic review and meta-analysis.**

# **Table S8: GRADE of quality evidence for antidiabetic medications on DR**

| **Comparison** | **Risk of Bias** | **Inconsistency** | **Indirectness** | **Imprecision** | **Publication bias** | **Quality** |
| --- | --- | --- | --- | --- | --- | --- |
| **Drugs classes** | | | | | | |
| Placebo | | | | | | |
| GLP-1 agonists vs Placebo | Not serious | Serious ^a^ | Not serious | Very Serious ^d^ | None | Very Low |
| SGLT2 inhibitors vs Placebo | Not serious | Serious ^a^ | Not serious | No serious | None | Moderate |
| DPP4 inhibitors vs Placebo | Not serious | Serious ^a^ | Not serious | Serious ^c^ | None | Low |
| All classes of antidiabetic medications | | | | | | |
| Meglitinides vs all classes of antidiabetic medications | Not serious | Not serious | Not serious | Extremely serious ^d,e^ | None | Very Low |
| SGLT2 inhibitors vs SU | Not serious | Serious ^a^ | Not serious | Very Serious ^d^ | None | Very low |
| TZD vs all classes of antidiabetic medications | Not serious | Not serious | Not serious | Very Serious ^d^ | None | Low |
| GLP-1 agonists vs all classes of antidiabetic medications | Not serious | Serious ^a^ | Not serious | Very Serious ^d^ | None | Very Low |
| DPP4 inhibitors vs all classes of antidiabetic medications | Not serious | Very Serious ^a,b^ | Not serious | Very Serious ^d^ | Reporting bias ^f^ | Very Low |
| Metformin vs all classes of antidiabetic medications | Not serious | Very Serious ^a,b^ | Not serious | Very Serious ^d^ | Reporting bias ^f^ | Very Low |
| SU vs all classes of antidiabetic medications | Not serious | Not serious | Not serious | Very Serious ^d^ | None | Low |
| Insulin vs all classes of antidiabetic medications | Not serious | Not serious | Not serious | Not serious | None | High ^g^ |
| Acarbose vs all classes of antidiabetic medications | Not serious | Not serious | Not serious | Extremely serious ^d,e^ | None | Very Low |
| **Individual drugs of GLP-1 agonists** | | | | | | |
| Placebo | | | | | | |
| Lixisenatide vs Placebo | Not serious | Not serious | Not serious | Not serious | None | High |
| Liraglutide vs Placebo | Not serious | Not serious | Not serious | Very Serious ^d^ | None | Low |
| Semaglutide vs Placebo | Not serious | Not serious | Not serious | Serious ^c^ | None | Moderate |
| Exenatide vs Placebo | Not serious | Not serious | Not serious | Very Serious ^d^ | None | Low |
| All classes of antidiabetic medications | | | | | | |
| Dulaglutide vs all classes of antidiabetic medications | Not serious | Not serious | Not serious | Very Serious ^d^ | None | Low |
| Liraglutide vs all classes of antidiabetic medications | Not serious | Serious ^a^ | Not serious | Very Serious ^d^ | None | Very low |
| Semaglutide vs all classes of antidiabetic medications | Not serious | Serious ^a^ | Not serious | Very Serious ^d^ | None | Very low |
| Exenatide vs all classes of antidiabetic medications | Not serious | Serious ^a^ | Not serious | Very Serious ^d^ | None | Very low |
| **Detailed drug comparisons** | | | | | | |
| SU | | | | | | |
| SU vs Acarbose | Not serious | Not serious | Not serious | Very Serious ^d^ | None | Low |
| SU vs TZD | Not serious | Not serious | Not serious | Serious ^c^ | None | Moderate |
| SU vs GLP-1 agonists | Not serious | Not serious | Not serious | Very Serious ^d^ | None | Low |
| SU vs SGLT2 inhibitors | Not serious | Serious ^a^ | Not serious | Very Serious ^d^ | None | Very low |
| SU vs DPP4 inhibitors | Not serious | Not serious | Not serious | Very Serious ^d^ | None | Low |
| SU vs Meglitinides | Not serious | Not serious | Not serious | Very Serious ^d^ | None | Low |
| Meglitinides | | | | | | |
| Meglitinides vs Acarbose | Not serious | Not serious | Not serious | Very Serious ^d^ | None | Low |
| Meglitinides vs SU | Not serious | Not serious | Not serious | Very Serious ^d^ | None | Low |
| DPP4 inhibitors | | | | | | |
| DPP4 inhibitors vs SU | Not serious | Not serious | Not serious | Very Serious ^d^ | None | Low |
| DPP4 inhibitors vs GLP-1 agonists | Not serious | Not serious | Not serious | Serious ^c^ | None | Low |
| DPP4 inhibitors vs Metformin | Not serious | Not serious | Not serious | Very Serious ^d^ | None | Low |
| GLP-1 agonists | | | | | | |
| GLP-1 agonists vs TZD | Not serious | Not serious | Not serious | Very Serious ^d^ | None | Low |
| GLP-1 agonists vs SU | Not serious | Not serious | Not serious | Very Serious ^d^ | None | Low |
| GLP-1 agonists vs Insulin | Not serious | Serious ^a^ | Not serious | Very Serious ^d^ | None | Very low |
| GLP-1 agonists vs DPP4 inhibitors | Not serious | Serious ^a^ | Not serious | Very Serious ^d^ | None | Low |
| SGLT2 inhibitors | | | | | | |
| SGLT2 inhibitors vs SU | Not serious | Serious ^a^ | Not serious | Very Serious ^d^ | None | Very low |
| TZD | | | | | | |
| TZD vs SU | Not serious | Not serious | Not serious | Very Serious ^d^ | None | Low |
| TZD vs Metformin | Not serious | Not serious | Not serious | Serious ^c^ | None | Moderate |
| TZD vs GLP-1 agonists | Not serious | Not serious | Not serious | Very Serious ^d^ | None | Low |
| Acarbose | | | | | | |
| Acarbose vs Meglitinides | Not serious | Not serious | Not serious | Very Serious ^d^ | None | Low |
| Acarbose vs SU | Not serious | Not serious | Not serious | Very Serious ^d^ | None | Low |
| Semaglutide | | | | | | |
| Semaglutide vs DPP4 inhibitors | Not serious | Not serious | Not serious | Very Serious ^d^ | None | Low |
| Semaglutide vs GLP-1 agonists | Not serious | Serious ^a^ | Not serious | Very Serious ^d^ | None | Very low |

**a, Estimated risk were different were differed in the direction between meta-analysis and primary studies;**

**b, Significant heterogeneity between these studies was observed (I^2^>50%);**

**c, Serious imprecision was considered if the 95% confidence intervals overlapped with the minimally important difference for clinical benefit (RR,0.75) or harm (RR>1.25)**

**d, Very serious imprecision was considered if the 95% confidence intervals include both clinically important benefit (RR<0.75) and harm (RR>1.25) or with borderline significance of benefit or harm**

**e, Few evidence or few patients;**

**f, Egger's P value less than 0.05;**

**g, large effect (RR<0.5 or RR>2).**

# **Table S9: Excluded systematic reviews**

| **1^st^ Author** | **Year** | **Title** | **Reason for Exclusion** |
| --- | --- | --- | --- |
| **Avgerinos I** | 2018 | Effect of GLP-1 receptor agonists on microvascular endpoints in type 2 diabetes: A systematic review and meta-analysis | Conference paper, the published article has included in this study |
| **Coon SA** | 2018 | Semaglutide once-weekly: improved efficacy with a new safety warning | Literature review |
| **Bignamini AA** | 2021 | Sulodexide for Diabetic-Induced Disabilities: A Systematic Review and Meta-Analysis | Included people with type 1 diabetes |
| **Miles KE** | 2018 | Semaglutide for the Treatment of Type 2 Diabetes Mellitus | Literature review |
| **Crosby-Nwaobi R** | 2012 | A systematic review of the association of diabetic retinopathy and cognitive impairment in people with Type 2 diabetes | No antidiabetic medications taken as an intervention or a comparator |
| **Valero MA** | 2011 | Meta-analysis on the role of lycopene in type 2 diabetes mellitus | No antidiabetic medications taken as an intervention or a comparator |
| **Tian C** | 2011 | Association of the C47T polymorphism in SOD2 with diabetes mellitus and diabetic microvascular complications: a meta-analysis | No antidiabetic medications taken as an intervention or a comparator |

# **Figure S1: The forest plot of the comparison within different drug classes**

***Note: * This meta-analysis result was reported by included reviews.***

Abbreviations: CI: confidence interval; ; GRADE=Grading of Recommendations, Assessment, Development and Evaluation; SGLT-2i: sodium-glucose cotransporter-2 inhibitors; GLP-1 RA: glucagon-like peptide-1 agonists; DPP-4i: dipeptidyl peptidase-4 inhibitors; NA: not available.

**Figure S2: The forest plot of Semaglutide**

Abbreviations: CI: confidence interval; ; GRADE=Grading of Recommendations, Assessment, Development and Evaluation; SGLT-2i: sodium-glucose cotransporter-2 inhibitors; GLP-1 RA: glucagon-like peptide-1 agonists; DPP-4i: dipeptidyl peptidase-4 inhibitors; NA: not available.

# **Table S10: AMSTAR2 items for systematic reviews and meta-analyses included in the umbrella review**

| **Study** | **Item No.** | | | | | | | | | | | | | | | | |
| --- | --- | --- | --- | --- | --- | --- | --- | --- | --- | --- | --- | --- | --- | --- | --- | --- | --- |
|  | 1 | 2 | 3 | 4 | 5 | 6 | 7 | 8 | 9 | 10 | 11 | 12 | 13 | 14 | 15 | 16 | Total AMSTAR2 Score |
| Wang, 2021^1^ | Yes | Partial yes | Yes | Partial yes | No | No | Partial yes | Partial yes | Yes | Yes | Yes | Yes | Yes | Yes | Yes | Yes | High |
| Tsapas, 2020^2^ | Yes | Yes | Yes | Partial yes | No | No | No | Partial yes | Yes | Yes | Yes | Yes | Yes | Yes | Yes | Yes | Low |
| Avgerinos, 2020^3^ | Yes | No | Yes | Partial yes | No | No | Partial yes | Partial yes | Yes | Yes | Yes | Yes | Yes | Yes | Yes | Yes | Low |
| Avgerinos, 2019^4^ | Yes | Partial yes | Yes | Partial yes | No | No | Yes | Partial yes | Yes | Yes | Yes | Yes | Yes | Yes | No | Yes | High |
| Tang, 2018^5^ | Yes | Yes | Yes | Partial yes | No | No | Partial yes | Partial yes | Yes | Yes | Yes | Yes | Yes | Yes | Yes | Yes | Moderate |
| Andreadis, 2018^6^ | Yes | Partial yes | Yes | Partial yes | No | No | Yes | Partial yes | Yes | Yes | Yes | Yes | Yes | Yes | Yes | Yes | High |
| Gargiulo, 2017^7^ | Yes | No | Yes | Partial yes | Yes | Yes | No | No | No | Yes | Yes | No | No | Yes | Yes | No | Critically Low |
| Li, 2021^8^ | Yes | No | Yes | Partial yes | No | No | Yes | Partial yes | Yes | Yes | Yes | Yes | Yes | Yes | Yes | Yes | Low |
| Bethel, 2021^9^ | Yes | Partial yes | Yes | Partial yes | Yes | Yes | No | Partial yes | No | Yes | Yes | No | No | No | No | Yes | Critically Low |
| Caparrotta, 2021^10^ | Yes | Partial yes | Partial yes | No | No | No | Yes | No | Yes | Yes | No meta-analysis conducted | No meta-analysis conducted | Yes | No | No meta-analysis conducted | Yes | High |
| Zhao, 2014  ^11^ | Yes | Yes | Yes | Partial yes | No | No | Partial yes | Partial yes | Yes | Yes | Yes | Yes | Yes | Yes | Yes | Yes | Moderate |

# **Table S11: Table of meta-analysis results before and after updating**

| **Drug Class** | **Study ID for preferred review** | **Results before update** | **Results after update** |
| --- | --- | --- | --- |
| **Comparing with Placebo** | | | |
| GLP-1agonist vs placebo | Avgerinos 2019 | 0.99 (0.87-1.13) | 0.98 (0.89-1.08) |
| SGLT2 inhibitors vs Placebo | Tang 2018 | 0.79 (0.55-1.13) | 1.00 (0.79-1.27) |
| DPP4 inhibitors vs Placebo | Tang 2018 | 1.23 (1.02-1.49) | 1.17 (0.99-1.39) |
| **Comparing with other antidiabetic drugs** | | | |
| GLP-1 agonist vs All classes of antidiabetic drugs | Avgerinos 2019 | 1.17 (0.83-1.64) | 0.93 (0.79-1.10) |
| GlP-1 agonist vs DPP4 inhibitors | Andreadis 2018 | 0.50 (0.18-1.41) | 1.00 (0.76-1.30) |
| GLP-1 agonist vs TZD | Tang 2018 | 0.60 (0.28-1.29) | 0.61 (0.30-1.22) |
| GLP-1 agonist vs SU | Avgerinos 2019 | 0.80 (0.47-1.36) | 0.84 (0.59-1.19) |
| DPP4 inhibitors vs SU | Tang 2018 | 0.58 (0.25-1.37) | 0.57 (0.25-1.28) |
| **Individual drugs of GLP-1 agonists** | | | |
| **Comparing with placebo** | | | |
| Liraglutide vs Placebo | Avgerinos 2019 | 1.22 (0.95-1.57) | 0.93 (0.62-1.39) |
| Semaglutide vs Placebo | Wang 2022 | 1.22 (1.01-1.47) | 1.23 (1.01-1.49) |
| **Comparing with other antidiabetic drugs** | | | |
| Liraglutide vs All other classes of antidiabetic drugs | Avgerinos 2019 | 1.06 (0.69-1.63) | 0.89 (0.63-1.25) |
| Semaglutide vs All other classes of antidiabetic drugs | Wang 2022 | 1.29 (1.02-1.64) | 1.05 (0.79-1.40) |
| Semaglutide vs DPP4 inhibitors | Andreadis 2018 | 0.50 (0.18-1.41) | 1.13 (0.77-1.66) |

Abbreviation: SGLT2 inhibitors: sodium-glucose cotransporter-2 inhibitors; GLP-1agonists: glucagon-like peptide-1 agonists; DPP-4 inhibitors: dipeptidyl peptidase-4 inhibitors; TZD: thiazolidinediones; SU: sulfonylureas.

# **Table S12: Quantitative synthesis, and bias assessment of 41 meta-analyses of antidiabetic drugs on DR**

| **Comparisons** | **Published meta-analyses** | | | | | **Individual studies** | | | | **Pooled analysis** | | | | **GRADE of evidence** |
| --- | --- | --- | --- | --- | --- | --- | --- | --- | --- | --- | --- | --- | --- | --- |
|  | Author | N. of patients | N. of studies | RR (95%CI) | I^2^ (%) | N. of patients | N of studies | RR (95%CI) | I^2^ (%) | N. of patients | N. of studies | RR (95%CI) | I^2^ (%) |  |
| **Drug classes** | | | | | | | | | | | | | | |
| Placebo | | | | | | | | | | | | | | |
| GLP-1 agonist vs Placebo | Avgerinos 2019 | 31624 | 15 | 0.99 (0.87-1.13) | 0% | 57194 | 22 | 1.06 (0.92-1.22) | 0% | 88818 | 37 | 0.98 (0.89-1.08) | 0% | Very Low |
| SGLT2 inhibitors vs Placebo | Tang 2018 | 7962 | 3 | 0.79 (0.55-1.13) | 0% | 25003 | 7 | 1.21 (0.88-1.67) | 0% | 32965 | 10 | 1.00 (0.79-1.27) | 0% | Moderate |
| DPP4 inhibitors vs Placebo | Tang 2018 | 39717 | 7 | 1.23 (1.02-1.49) | 0% | 10713 | 5 | 0.83 (0.55-1.25) | 0% | 50430 | 12 | 1.17 (0.99-1.39) | 0% | Low |
| **All classes of antidiabetic medications** | | | | | | | | | | | | | | |
| Meglitinides vs all other classes of antidiabetic medications | NA | NA | NA | NA | NA | 2740 | 2 | 0.34 (0.10-8.25) | 0% | 2740 | 2 | 0.34 (0.10-8.25) | 0% | Very Low |
| SU vs SGLT2 inhibitors | Tang 2018 | 2999 | 2 | 1.37 (0.19-9.74) | 69.1% | NA | NA | NA | NA | 2995 | 2 | 0.73 (0.10-5.16) | 69.1% | Very low |
| TZD vs all other classes of antidiabetic medications | NA | NA | NA | NA | NA | 4995 | 3 | 0.92 (0.67-1.26) | 30% | 4995 | 3 | 0.92 (0.67-1.26) | 30% | Low |
| GLP-1 agonists vs all other classes of antidiabetic medications | Avgerinos 2019 | 11193 | 18 | 1.17 (0.83-1.64) | 0% | 15192 | 11 | 0.91 (0.68-1.21) | 0% | 26385 | 39 | 0.93 (0.79-1.10) | 0% | Very Low |
| DPP4 inhibitors vs all other classes of antidiabetic medications | Tang 2018 | 1031 | 2 | 0.58 (0.25-1.37) | 1% | 16272 | 15 | 1.41 (0.55-3.57) | 67% | 17303 | 17 | 0.93 (0.74-1.18) | 63% | Very Low |
| Metformin vs all other classes of antidiabetic medications | NA | NA | NA | NA | NA | 2888 | 2 | 1.15 (0.81-1.63) | 0% | 2888 | 2 | 1.15 (0.81-1.63) | 0% | Low |
| SU vs all other classes of antidiabetic medications | NA | NA | NA | NA | NA | 17865 | 15 | 1.28 (0.96-1.71) | 0% | 17865 | 15 | 1.24 (0.93-1.65) | 0% | Moderate |
| Insulin vs all other classes of antidiabetic medications | Zhao 2014 | 20818 | 7 | 2.30 (1.35-3.93) | 1% | NA | NA | NA | NA | 20818 | 7 | 2.47 (2.04-2.99) | 53% | High |
| Acarbose vs all other classes of antidiabetic medications | NA | NA | NA | NA | NA | 2746 | 2 | 4.21 (0.44-40.43) | 0% | 2746 | 2 | 4.21(0.44-40.43) | 0% | Very Low |
| **Individual drugs of GLP-1 agonists** | | | | | | | | | | | | | | |
| Placebo | | | | | | | | | | | | | | |
| Lixisenatide vs Placebo | Avgerinos 2019 | 484 | 1 | 0.90 (0.75-1.09) | NA | NA | NA | NA | NA | 484 | 1 | 0.90 (0.75-1.09) | NA | High |
| Liraglutide vs Placebo | Avgerinos 2019 | 12725 | 11 | 1.22 (0.95-1.57) | 0% | 426 | 1 | 1.00 (0.19-5.39) | 0% | 13151 | 12 | 0.93 (0.62-1.39) | 0% | Low |
| Semaglutide vs Placebo | Wang 2021 | 11099 | 10 | 1.22 (1.01-1.47) | 0% | 779 | 1 | 0.52 (0.02-12.58) | 0% | 11878 | 11 | 1.23 (1.01-1.49) | 0% | Moderate |
| Exenatide vs Placebo | Avgerinos 2019 | 484 | 1 | 1.50 (0.06-36.62) | NA | NA | NA | NA | NA | 484 | 1 | 1.50 (0.06-36.62) | NA | Low |
| All classes of antidiabetic medications | | | | | | | | | | | | | | |
| Liraglutide vs all classes of antidiabetic medications | Avgerinos 2019 | 5050 | 9 | 1.06 (0.69-1.63) | 0% | 3972 | 7 | 1.79 (0.57-5.63) | 11% | 9022 | 16 | 0.89 (0.63-1.25) | 0% | Very low |
| Dulaglutide vs all classes of antidiabetic medications | Avgerinos 2019 | 1934 | 3 | 0.95 (0.23-3.96) | 0% | NA | NA | NA | NA | 1934 | 3 | 0.95 (0.23-3.96) | 0% | Low |
| Semaglutide vs all classes of antidiabetic medications | Wang 2021 | 11238 | 15 | 0.98 (0.77-1.26) | 0% | 1681 | 2 | 0.59 (0.04-9.30) | 81% | 12919 | 17 | 1.05 (0.79-1.40) | 20% | Very low |
| Exenatide vs all classes of antidiabetic medications | Avgerinos 2019 | 994 | 2 | 6.32 (0.30-131.26) | 0% | NA | NA | NA | NA | 994 | 2 | 6.32 (0.30-131.26) | 0% | Very low |
| **Detailed drug comparisons** | | | | | | | | | | | | | | |
| SU | | | | | | | | | | | | | | |
| SU vs Acarbose | Tang 2018 | 1096 | 1 | 0.17 (0.01-4.10) | 0% | NA | NA | NA | NA | 1096 | 1 | 0.17 (0.01-4.10) | 0% | Low |
| SU vs TZD | Tang 2018 | 2222 | 1 | 1.24 (0.84-1.83) | 0% | NA | NA | NA | NA | 2222 | 1 | 1.24 (0.84-1.83) | 0% | Moderate |
| SU vs SGLT2 inhibitors | Tang 2018 | 2995 | 2 | 1.37 (0.19-9.74) | 69.1% | NA | NA | NA | NA | 2995 | 2 | 1.37 (0.19-9.74) | 69.1% | Very low |
| SU vs GLP-1 agonist | Tang 2018 | 1814 | 3 | 0.80 (0.47-1.36) | 0% | 4157 | 6 | 1.25 (0.66-2.38) | NA | 5971 | 9 | 1.41 (1.00-2.00) | 0% | Low |
| SU vs DPP4 inhibitors | Tang 2018 | 1031 | 2 | 1.73 (0.73-4.08) | 12.1% | 6033 | 1 | 2.01 (0.18-22.14) | NA | 7064 | 3 | 1.78 (0.79-4.02) | 0% | Low |
| SU vs Meglitinides | Tang 2018 | 1644 | 1 | NA | 0% | NA | NA | NA | NA | 1644 | 1 | NA | NA | Low |
| Meglitinides | | | | | | | | | | | | | | |
| Meglitinides vs Acarbose | Tang 2018 | 1096 | 1 | 0.34 (0.01-8.25) | 0% | NA | NA | NA | NA | 1096 | 1 | 0.34 (0.01-8.25) | 0% | Low |
| Meglitinides vs SU | Tang 2018 | 1644 | 1 | NA | 0% | NA | NA | NA | NA | 1644 | 1 | NA | 0% | Low |
| Metformin | | | | | | | | | | | | | | |
| Metformin vs DPP4 inhibitors | Tang 2018 | 663 | 1 | 0.34 (0.01-8.33) | NA | NA | NA | NA | NA | 663 | 1 | 0.34 (0.01-8.33) | NA | Low |
| Metformin vs TZD | Tang 2018 | 2225 | 1 | 1.17 (0.82-1.66) | NA | NA | NA | NA | NA | 2225 | 1 | 1.17 (0.82-1.66) | NA | Low |
| DPP4 inhibitors | | | | | | | | | | | | | | |
| DPP4 inhibitors vs SU | Tang 2018 | 1031 | 2 | 0.58 (0.25-1.37) | 12.10% | 6033 | 1 | 0.50 (0.05-5.49) | NA | 7064 | 3 | 0.57 (0.25-1.28) | 0% | Low |
| DPP4 inhibitors vs GLP-1 agonist | Tang 2018 | 9576 | 3 | 0.86 (0.48-1.55) | 0% | 7819 | 10 | 0.82 (0.62-1.07) | 2% | 9576 | 13 | 0.82 (0.64-1.05) | 0% | Low |
| DPP4 inhibitors vs Metformin | Tang 2018 | 663 | 1 | 2.94 (0.72-71.85) | 0% | NA | NA | NA | NA | 663 | 1 | 2.94 (0.72-71.85) | 0% | Low |
| GLP-1 agonist | | | | | | | | | | | | | | |
| GLP-1 agonist vs TZD | Tang 2018 | 548 | 1 | 0.60 (0.28-1.29) | 0% | 278 | 1 | 0.64 (0.11-3.76) | 0% | 826 | 2 | 0.61 (0.30-1.22) | 0% | Low |
| GLP-1 agonist vs Insulin | Tang 2018 | 2299 | 4 | 0.96 (0.64-1.44) | 0% | 4451 | 8 | 0.67 (0.35-1.26) | 0% | 6750 | 12 | 0.83 (0.59-1.16) | 0% | Very low |
| GLP-1 agonist vs SU | Tang 2018 | 1814 | 3 | 0.80 (0.47-1.36) | 0% | 4160 | 4 | 0.80 (0.42-1.51) | 0% | 5971 | 9 | 0.84 (0.59-1.19) | 0% | Low |
| GLP-1 agonist vs DPP4 inhibitors | Tang 2018 | 1757 | 3 | 1.16 (0.64-2.09) | 0% | 7819 | 10 | 0.97 (0.74-1.28) | 0% | 9576 | 13 | 1.05 (0.82-1.34) | 0% | Low |
| SGLT2 inhibitors | | | | | | | | | | | | | | |
| SGLT2 inhibitors vs SU | Tang 2018 | 2995 | 2 | 0.73 (0.10-5.16) | 69.10% | NA | NA | NA | NA | 2995 | 2 | 0.73 (0.10-5.16) | 0% | Very low |
| TZD | | | | | | | | | | | | | | |
| TZD vs SU | Tang 2018 | 2222 | 1 | 0.81 (0.55-1.19) | 0% | NA | NA | NA | NA | 2222 | 1 | 0.81 (0.55-1.19) | 0% | Low |
| TZD vs Metformin | Tang 2018 | 2225 | 1 | 0.86 (0.60-1.22) | 0% | NA | NA | NA | NA | 2225 | 1 | 0.86 (0.60-1.22) | 0% | Moderate |
| TZD vs GLP-1 agonist | Tang 2018 | 548 | 1 | 1.66 (0.78-3.57) | 0% | 278 | 1 | 1.57 (0.27-9.23) | 0% | 826 | 2 | 1.67 (0.83-3.36) | 0% | Low |
| Acarbose | | | | | | | | | | | | | | |
| Acarbose vs Meglitinides | Tang 2018 | 1096 | 1 | 2.97 (0.12-72.68) | 0% | NA | NA | NA | NA | 1096 | 1 | 2.97 (0.12-72.68) | 0% | Low |
| Acarbose vs SU | Tang 2018 | 1650 | 1 | 5.98 (0.24-146.51) | 0% | NA | NA | NA | NA | 1650 | 1 | 5.98 (0.24-146.51) | 0% | Low |
| Semaglutide | | | | | | | | | | | | | | |
| Semaglutide vs other GLP-1 agonist | Andreadis 2018 | 1385 | 2 | 0.50 (0.15-1.66) | 9% | 2117 | 4 | 1.41 (0.72-2.76) | 0% | 3502 | 6 | 1.09 (0.58-2.03) | 0% | Very low |
| Semaglutide vs DPP4 inhibitors | Andreadis 2018 | 1533 | 2 | 0.50 (0.18-1.41) | 23% | 4472 | 5 | 1.21 (0.84-1.76) | 21% | 6005 | 7 | 1.13 (0.77-1.66) | 26% | Low |

Abbreviation: SGLT2 inhibitors: sodium-glucose cotransporter-2 inhibitors; GLP-1agonists: glucagon-like peptide-1 agonists; DPP-4 inhibitors: dipeptidyl peptidase-4 inhibitors; TZD: thiazolidinediones; SU: sulfonylureas.

# **Figure S3:Forest plot of SGLT-2 inhibitors vs Placebo**

# **Figure S4:Forest plot of GLP-1 agonists vs Placebo**

# **Figure S5: Forest plot of DPP4 inhibitors vs Placebo**

# **Figure S6: Forest plot of Meglitinides vs All other classes of antidiabetic drugs**

# **Figure S7: Forest plot of TZD vs All other classes of antidiabetic drugs**

# **Figure S8: Forest plot of GLP-1 agonists vs All other classes of antidiabetic drugs**

# **Figure S9: Forest plot of DPP4 inhibitors vs All other classes of antidiabetic drugs**

# **Figure S10: Forest plot of SU vs All other classes of antidiabetic drugs**

# **Figure S11: Forest plot of Acarbose vs All other classes of antidiabetic drugs**

# **Figure S12: Forest plot of Metformin vs All other classes of antidiabetic drugs**

# **Figure S13: Forest plot of Semaglutide vs Placebo**

# **Figure S14: Forest plot of Semaglutide vs All other classes of antidiabetic drugs**

# **Figure S15: Forest plot of Semaglutide vs DPP-4 inhibitors**

# **Figure S16: Forest plot of Semaglutide vs other GLP-1 agonists**

# **Figure S17: Forest plot of Liraglutide vs Placebo**

# **Figure S18: Forest plot of Liraglutide vs All other classes of antidiabetic drugs**

**Figure S19: Forest plot of GLP-1 vs SU**

**Figure S20: Forest plot of GLP-1 vs TZD**

**Figure S21: Forest plot of GLP-1 vs Insulin**

**Figure S22: Forest plot of GLP-1 vs DPP4**

**Figure S23: Funnel plot of SGLT-2 inhibitors vs Placebo**

# **Figure S24: Funnel plot of GLP-1 agonists vs Placebo**

# **Figure S25: Funnel plot of DPP4 inhibitors vs Placebo**

# **Figure S26: Funnel plot of GLP-1 agonists vs All other classes of antidiabetic drugs**

# **Figure S27: Funnel plot of DPP4 inhibitors vs All other classes of antidiabetic drugs**

# **Figure S28: Funnel plot of SU vs All other classes of antidiabetic drugs**

**Figure S29: Funnel plot of Semaglutide vs Placebo**

# **Figure S30: Funnel plot of Semaglutide vs All other classes of antidiabetic drugs**

# **Figure S31: Funnel plot of Liraglutide vs Placebo**

# **Figure S32: Funnel plot of Liraglutide vs All other classes of antidiabetic drugs**

# **Figure S33: Funnel plot of GLP-1 agonists vs TZD**

# **Figure S34: Funnel plot of GLP-1 agonists vs Insulin**

# **Figure S35: Funnel plot of GLP-1 agonists vs DPP4**

# **Figure S36: Funnel plot of GLP-1 agonists vs SU**
